# Structural and biochemical characterization of *Grimontia hollisae* thermostable direct hemolysin with DNA reveals first *Vibrio* hemolysin with nuclease activity

**DOI:** 10.1093/nar/gkag679

**Published:** 2026-07-06

**Authors:** Po-Yun Hsiao, Yu-Kuo Wang, Sheng-Cih Huang, Feng-Pai Chou, Tzu-Yu Huang, Yen-Cheng Lin, You-Min Kuo, Tung-Kung Wu, Chin-Yuan Chang

**Affiliations:** Department of Biological Science and Technology, National Yang Ming Chiao Tung University, Hsinchu 30010 Taiwan, Republic of China; Department of Biological Science and Technology, National Yang Ming Chiao Tung University, Hsinchu 30010 Taiwan, Republic of China; Department of Biological Science and Technology, National Yang Ming Chiao Tung University, Hsinchu 30010 Taiwan, Republic of China; Department of Biological Science and Technology, National Yang Ming Chiao Tung University, Hsinchu 30010 Taiwan, Republic of China; Department of Biological Science and Technology, National Yang Ming Chiao Tung University, Hsinchu 30010 Taiwan, Republic of China; Department of Biological Science and Technology, National Yang Ming Chiao Tung University, Hsinchu 30010 Taiwan, Republic of China; Department of Biological Science and Technology, National Yang Ming Chiao Tung University, Hsinchu 30010 Taiwan, Republic of China; Department of Biological Science and Technology, National Yang Ming Chiao Tung University, Hsinchu 30010 Taiwan, Republic of China; Center for Emergent Functional Matter Science, National Yang Ming Chiao Tung University, Hsinchu 30010 Taiwan, Republic of China; Center for Intelligent Drug Systems and Smart Biodevices (IDS2B), National Yang Ming Chiao Tung University, Hsinchu 30010 Taiwan, Republic of China; Department of Biological Science and Technology, National Yang Ming Chiao Tung University, Hsinchu 30010 Taiwan, Republic of China; Center for Emergent Functional Matter Science, National Yang Ming Chiao Tung University, Hsinchu 30010 Taiwan, Republic of China; Center for Intelligent Drug Systems and Smart Biodevices (IDS2B), National Yang Ming Chiao Tung University, Hsinchu 30010 Taiwan, Republic of China; Department of Biomedical Science and Environmental Biology, Drug Development and Value Creation Research Center, Kaohsiung Medical University, Kaohsiung 807 Taiwan, Republic of China

## Abstract

*Grimontia hollisae* thermostable direct hemolysin (Gh-TDH) is a pore-forming toxin that disrupts the cell membrane, leading to erythrocyte lysis and cytotoxicity. Previous studies showed that fluorescently labeled Gh-TDH-FITC binds to hepatocyte membranes and subsequently translocates to the nucleus. Here, we demonstrate for the first time that Gh-TDH cleaves DNA with 3**′**–5**′** nuclease activity. The crystal structure of Gh-TDH in complex with ssDNA unveils a unique DNA-binding configuration. Notably, the putative cleavage site (Tyr87-Lys88-Asp89) deviates from the canonical 3**′**–5**′** exonuclease motif (Asp-Glu-Asp-Asp). Site-directed mutagenesis and binding kinetics assays demonstrate that Lys88 is essential for nuclease activity, supporting its central role in catalysis. TDH homologues are widespread in *Vibrio*, with DNA-binding and catalytic residues highly conserved. Consistently, *V. parahaemolyticus* TDH also exhibits 3′–5′ exonuclease activity, indicating this nuclease function is an intrinsic, conserved feature of the TDH family rather than incidental. These findings provide the structural basis and mechanism of DNA binding and cleavage by Gh-TDH. Gh-TDH is thus the first *Vibrio* pore-forming toxin shown to have dual hemolytic and nuclease activities. This work opens new avenues to explore why *Vibrio* pore-forming toxins have evolved nuclease activity and what physiological or pathogenic roles it may play *in vivo*.

## Graphical Abstract

**Graphical Abstract:**
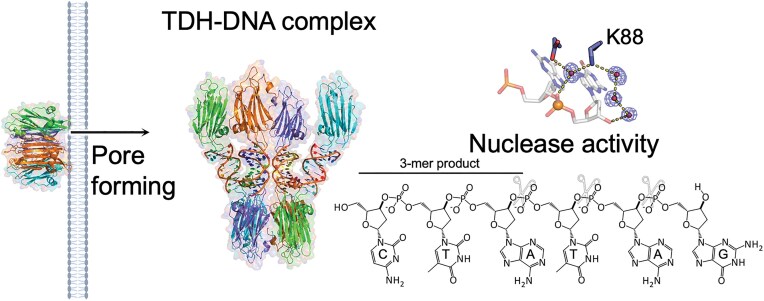

## Introduction

Pathogenic bacteria produce a range of toxins, including pore-forming toxins (PFTs), cytotoxins, neurotoxins, superantigens, and nucleases, to damage host cells and evade immune responses. For example, *Vibrio cholerae* cytolysin disrupts cell membranes, causing severe gastrointestinal symptoms, while *Streptococcus pneumoniae* DNase degrades neutrophil extracellular traps (NETs), aiding immune evasion [1–3]. Group A *Streptococcus* (GAS) DNase exacerbates disease by degrading host DNA, reducing pus viscosity, and facilitating bacterial spread [4]. Similarly, *Mycoplasma bovis* MbovNase degrades NETs and induces apoptosis [5]. Notably, colicin E9 from *Escherichia coli* is the first identified bacterial PFT with nuclease activity, enabling DNA damage in the host cytoplasm [6, 7].

Thermostable direct hemolysin (TDH) is a PFT and a major virulence factor of pathogenic *Vibrio* species, including *V. parahaemolyticus, V. cholerae* non-O1, *V. mimicus, V. alginolyticus*, and *Grimontia hollisae* (formerly described as *V. hollisae*) etc. [8–13]. Ingesting raw seafood or exposure to wounds contaminated with *Vibrio* TDH can lead to hypovolemic shock, bacteremia, and sepsis [14–22]. Studies have shown that TDH from *G. hollisae* (Gh-TDH) induces osmotic lysis of red blood cells [23, 24], and exhibits cyto-, cardio-, entero-, and hepato-toxicity in cultured cells, as well as lethality in mice [16, 24–30]. Interestingly, while Gh-TDH binds erythrocyte membranes across species with similar affinity, its hemolytic activity varies, suggesting additional activities or factors contribute to its pathogenicity [31]. Additionally, a paradoxical phenomenon previously observed in Vp-TDH, known as the Arrhenius effect, was also observed in Gh-TDH [24, 32, 33]. This effect involves the suppression of hemolytic activity at 60–70°C, followed by reactivation at temperatures above 80°C, suggesting the existence of multiple protomer interfaces involved in its oligomerization. Crystal structure analysis reveals three distinct tetrameric oligomeric conformations of Gh-TDH: oligomer I, similar to *V. parahaemolyticus* (Vp-TDH), and oligomers II and III, which share a dimer motif. This dimer likely represents the minimal structural unit required for membrane binding and hemolytic activity, highlighting the structural complexity of Gh-TDH’s role in pathogenesis [30].

In a previous study, incubation of fluorescently labeled recombinant Gh-TDH-FITC with liver cells demonstrated that Gh-TDH binds to the cell membrane, induces extensive membrane blebbing, translocates to the nucleus, and ultimately triggers cell lysis and death [16]. This cytotoxic mechanism resembles the mode of action of glucocorticoids and certain toxic compounds, which, after entering the cytoplasm, translocate to the nucleus and bind to receptors to regulate gene expression, thereby activating endonucleases to induce apoptosis [34–37]. These findings suggest that Gh-TDH may enhance its cytotoxicity and pathogenicity either by activating endogenous nuclease-dependent apoptosis pathways or by directly cleaving DNA through its intrinsic nuclease activity.

In this study, *in vitro* nuclease activity assays and X-ray crystallography revealed the structural and mechanistic basis of Gh-TDH’s nuclease activity. The assays demonstrated that Gh-TDH binds DNA and catalyzes 3**′**–5**′** exonucleolysis, producing a 3-mer product. Structural analysis of the Gh-TDH-DNA complexes revealed a unique DNA-binding and cleavage mechanism, as Gh-TDH lacks the canonical catalytic motifs typical of 3**′**–5**′** exonucleases, highlighting its distinct nuclease properties [38]. This newly identified nuclease activity not only provides insights into why TDH translocates to the nucleus following its association with the cell membrane but also offers a new conceptual framework for understanding the multifunctional roles and pathogenic mechanisms of pore-forming toxin.

## Material and methods

### Molecular cloning, mutagenesis, gene expression, protein production, and purification of the recombinant Gh-TDH wild-type and the mutant variants

The Gh-TDH gene (GenBank accession ID: WP_040528653.1) was cloned into the pCR2.1-TOPO vector. For the mutant variants, the plasmids were constructed by the QuikChange site-directed mutagenesis method. All plasmids were confirmed via DNA sequencing. Each of the wild-type and the mutant constructs was transformed into *E. coli* BL21(DE3)pLysS cells for gene expression. Gene expression, protein production, and purification were carried out using previously established methods, yielding highly purified proteins for biochemical and structural analysis [24, 29]. The protein purity was assessed by SDS-PAGE. All wild-type and mutant variants of Gh-TDH proteins were concentrated using an Amicon Ultra-15 3 000 NMWL concentrator (Merck) in 20 mM Tris-HCl at pH 7.0 for the following nuclease activity assay, BLI analysis, and protein crystallization.

### Nuclease activity assay

Fluorescein amidite (FAM)-labeled DNA substrates (3**′**- or 5**′**-end) were synthesized by Genomics Inc., Taiwan, for nuclease activity assays. The sequences of DNA substrates used in nuclease activity assays are listed in Supplementary Table S1. In nuclease activity assays, 0.5 μM DNA substrates were incubated with protein in 5 mM MgCl_2_ and 20 mM Tris-HCl (pH 7.0). After incubation at 37 °C for 60 min, 2x TBE-urea loading buffer (G-Biosciences) was added to quench the reaction at 95 °C for 5 min. DNA digestion patterns were analyzed using 20% TBE urea polyacrylamide gels and visualized under blue light. Nuclease-specific activity was measured using 5 μM protein with 0.5 μM substrate, with excision quantified by analyzing the decrease in substrate band intensity using ImageJ.

To evaluate the nuclease activity of Gh-TDH toward ssDNA substrates containing 3′-end or 5′-end biotin–streptavidin modifications, four 20-mer linear ssDNA substrates with different terminal modifications were synthesized, including 5**′**-FAM-labeled ssDNA, 5**′**-FAM-/3**′**-biotin-labeled ssDNA, 3**′**-FAM-labeled ssDNA, and 3**′**-FAM-/5**′**-biotin-labeled ssDNA (Supplementary Table S1, entries 9, 11–13). Prior to the nuclease activity assay, four ssDNA substrates were incubated with streptavidin at room temperature for 30 min to allow formation of the biotin–streptavidin complex. Nuclease activity assays were then performed following the general procedure described above.

To evaluate the nuclease activity of Gh-TDH toward circular and linear ssDNA substrates, a 66-mer circular ssDNA and its corresponding linear ssDNA counterpart were synthesized (Sangon Biotech) based on a circular ssDNA sequence design reported previously [39] (Supplementary Table S1, entries 14 and 15). Nuclease activity assays were performed following the general procedure described above. Neither substrate was fluorescently labeled. DNA substrates and cleavage products were therefore detected by SYBR Gold (Invitrogen™) staining after gel electrophoresis.

### Enzyme kinetics of Gh-TDH

Exonuclease activity of Gh-TDH was assayed using 5′-FAM-labeled poly(dA₁₂), poly(dT₁₂), and poly(dC₁₂) DNA substrates (Supplementary Table S1). Reactions (10 μL) were performed in buffer containing 20 mM Tris–HCl (pH 7.0) and 5 mM MgCl₂. Gh-TDH was used at a final concentration of 5 μM, and reactions were initiated by the addition of DNA substrates at varying concentrations (0.25–6 μM). Samples were incubated at 37°C and terminated by addition of 2 × TBE–urea loading buffer followed by heating at 95°C for 5 min. Reaction products were separated on 20% denaturing polyacrylamide gels (urea–PAGE) and visualized. Band intensities corresponding to the substrate (12-mer) and digestion products (11-mer to 3-mer) were quantified using ImageJ. Intensities were normalized to the total signal within each lane to obtain fractional distributions, which were converted to concentrations by multiplying by the initial substrate concentration. To account for the processive nature of exonuclease activity, the total number of nucleotides removed was calculated as a weighted sum of all product species, where each product length reflects the number of nucleotides excised from the original 12-mer substrate. The initial velocity (*V*_0_​) was calculated as the total number of nucleotides removed divided by reaction time, yielding rates of nucleotide excision (μM·min⁻¹). Initial velocities measured at varying substrate concentrations were fitted to the Michaelis–Menten equation using GraphPad Prism 10. All reactions were carried out in triplicate.

### Crystallization, data collection, and structure determination

The purified Gh-TDH wild-type and K88A variant were mixed with ssDNA at a 1:1.3 molar ratio, respectively, and incubated on ice for 20 min. The mixtures were crystallized using the hanging-drop vapor-diffusion method at 20°C. The crystallization conditions and the sequences of co-crystallized ssDNA for complexes I–VI were listed in Supplementary Table S2. Crystals were cryo-protected with Paraton-N (Hampton Research) for data collection at BL15-A1, TPS-05A, and TPS-07A (NSRRC, Taiwan, R.O.C.). All diffraction data were indexed and scaled with HKL2000 [40]. Molecular replacement was performed using Gh-TDH (PDB entry: 4WX3) as the search model [30] in MOLREP of CCP4 [41]. Models were built in COOT [42] and refined with REFMAC [43]. The atomic coordinates and structure factors of complexes I–VI have been deposited in the Protein Data Bank with the accession code 9LCS, 9L8E, 9L9M, 9LCM, 9LCU, and 9LCW, respectively. Data processing and refinement statistics are summarized in Supplementary Table S3.

### Dynamic light scattering (DLS) analysis

Samples were prepared at a Gh-TDH concentration of 1 mg/mL and incubated with DNA (CTCTATAGAG) at molar ratios of 1:0.5, 1:1.25, and 1:2.5. Samples were equilibrated at 25°C prior to measurement. The size of Gh-TDH and Gh-TDH-DNA complexes was measured using a Zetasizer Nano ZS90 (Malvern Instruments, UK). Particle size was reported as the Z-average diameter.

### Job plot analysis

Job plot was performed in a general method [44, 45]. The total concentration of Gh-TDH and DNA substrate (Supplementary Table S1, entry 16) was maintained at 5 μM, while the molar ratio of Gh-TDH protomer to DNA was varied (1:8, 1:6, 1:4, 1:2, 1:1, 2:1, 4:1, 6:1, and 8:1). The resulting nuclease activity was quantified by measuring the concentration of nucleotide products. The total product concentration $( {{{C}_{{\mathrm{product}}}}} )$ was calculated by weighting the fluorescence intensity of each product band by the corresponding number of cleavage events, normalizing to the total fluorescence intensity of the remaining substrate and all products, and multiplying by the initial substrate concentration according to the following equation:


\begin{eqnarray*}
{{C}_{\textit{product}}} = \frac{{\sum\nolimits_i^9 {{{1}^{i{{I}_i}}}} }}{{{{I}_{\textit{total}}}}} \times {{C}_{\textit{substrate}}}
\end{eqnarray*}

where ${{I}_i}$ represents the fluorescence intensity of the product band corresponding to *i* cleavage events, *i* denotes the number of cleavage events represented by the product (*i* = 1–9), ${{C}_{{\mathrm{substrate}}}}$ is the initial substrate concentration, ${{I}_{{\mathrm{total}}}}$ is the sum of the fluorescence intensities of the remaining substrate and all product bands, and ${{C}_{{\mathrm{product}}}}$ is the total product concentration.

### Mass spectrometric analysis of Gh-TDH DNA cleavage products

Mass spectrometric analyses were performed by the Center for Advanced Instrumentation and Department of Applied Chemistry at National Yang Ming Chiao Tung University, Hsinchu, Taiwan, R.O.C. To analyze the molecular mass of DNA products cleaved by Gh-TDH, a 6-mer DNA substrate (CTATAG) (50 μM) was incubated with Gh-TDH (50 μM) in 20 mM Tris-HCl (pH 7.0) and 5 mM MgCl_2_ at 37°C for 4 h. To quench the reaction, the mixture was heated to 60°C for 10 min. The reaction mixture was centrifuged at 13 000 rpm to remove the fibrillar Gh-TDH, and the supernatant was subjected to HD Q-TOF with ESI(+)MS analysis (Bruker). The collected data were analyzed using Compass DataAnalysis 4.1 (Bruker).

### Bio-layer interferometry (BLI) binding kinetics assay

The binding affinity of 5**′**-biotinylated stem-loop dsDNA to Gh-TDH wild-type or the K88A variant was measured using the Octet HTX system (ForteBio) at the Center for Emergent Functional Matter Science, National Yang Ming Chiao Tung University, Taiwan, R.O.C. BLI measurements were carried out at a shaking speed of 1 000 rpm. The purified Gh-TDH proteins at various concentrations were prepared in kinetic buffer (PBS at pH 7.4, 50 mM EDTA, 1 mg/mL BSA) and loaded onto HIS1K (streptavidin) biosensors (Molecular Devices, ForteBio). Five concentrations of Gh-TDH in kinetic buffer were added to a black polypropylene 96-well microplate (Greiner Bio-one), with one row containing kinetic buffer as a reference control. Each protein concentration underwent an assay cycle using streptavidin-bound 5**′**-biotinylated stem-loop dsDNA or streptavidin-bound 5**′**-biotinylated poly(dA₁₂), poly(dT₁₂), or poly(dC₁₂) DNA probes, together with the corresponding blank probes. One assay cycle consists of 60 s of baseline normalization in kinetics buffer, 400 s of association in the protein solution, 200 s of dissociation in kinetics buffer, and 30 s of regeneration in 0.5 M NaOH. BLI results were analyzed using FortéBio Data Analysis High Throughput 12.0. The sequences of ssDNA substrates used in BLI assays are listed in Supplementary Table S1.

## Results

### Characterization of 3′–5′ exonuclease activity of Gh-TDH

**Figure 1.**
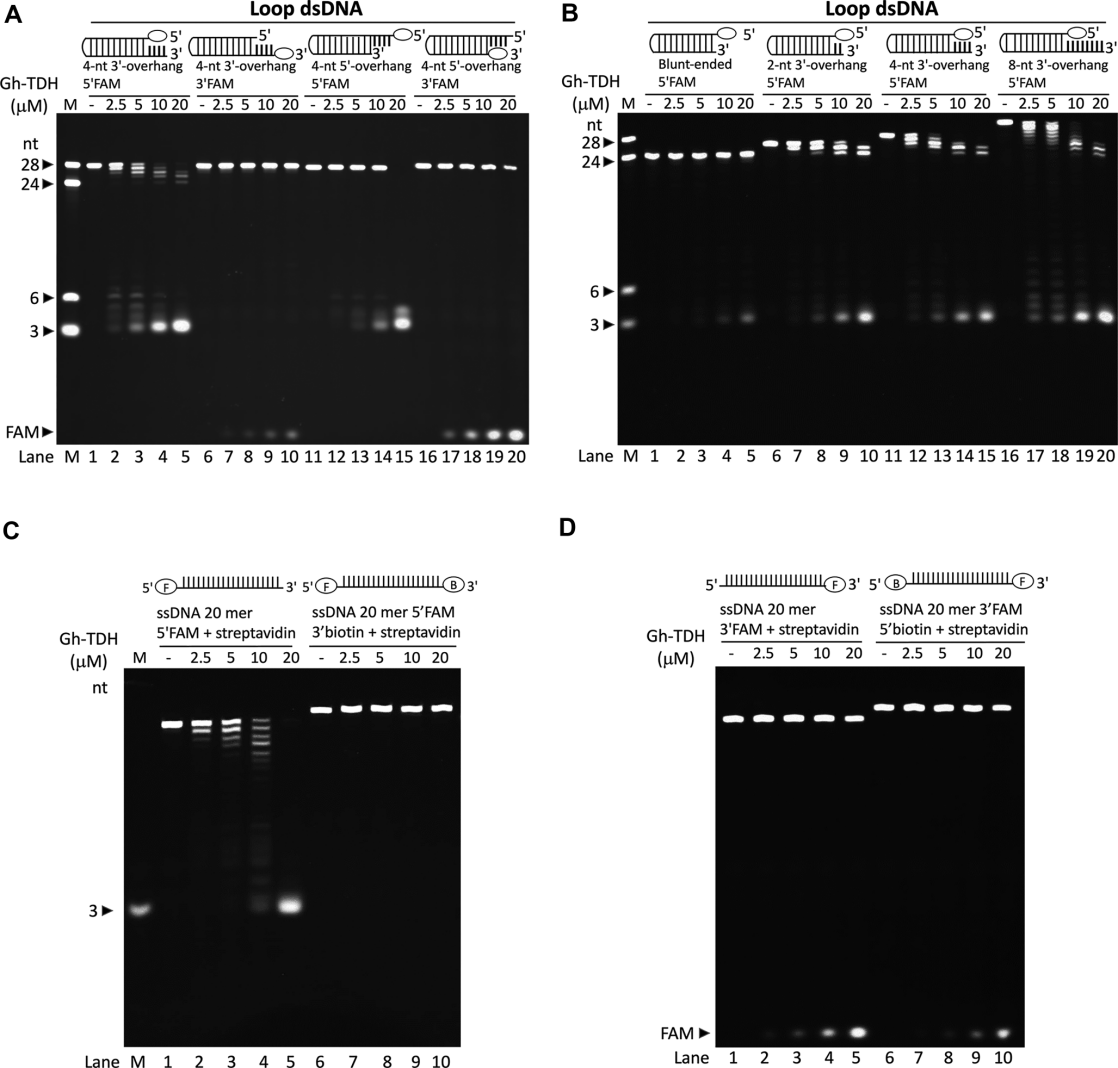
Nuclease activity assay of Gh-TDH. (**A**), Gh-TDH was incubated with 0.5 μM FAM-labeled stem-loop dsDNA substrates, including 5′-FAM-labeled 4-nt 3′-overhang stem-loop dsDNA (Lanes 1–5), 3′-FAM-labeled 4-nt 3′-overhang stem-loop dsDNA (Lanes 6–10), 5′-FAM-labeled 4-nt 5′-overhang stem-loop dsDNA (Lanes 11–15), and 3′-FAM-labeled 4-nt 5′-overhang stem-loop dsDNA (Lanes 16–20). (**B**), Gh-TDH was incubated with 0.5 μM 5′-FAM-labeled stem-loop dsDNA with varying 3′-end overhangs: blunt-ended dsDNA (Lanes 1–5), 2-nt 3′-overhang (Lanes 6–10), 4-nt 3′-overhang (Lanes 11–15), and 8-nt 3′-overhang (Lanes 16–20). Lane M represents a DNA marker of 28, 24, 6, and 3 nt. (**C**), Gh-TDH was incubated with streptavidin and 0.5 μM 5′-FAM-labeled ssDNA substrates with or without a 3′-biotin modification (Lanes 1–5 and Lanes 6–10). (**D**), Gh-TDH was incubated with streptavidin and 0.5 μM 3′-FAM-labeled ssDNA substrates with or without a 5′-biotin modification (Lanes 1–5 and Lanes 6–10).

Recombinant wild-type and mutant Gh-TDH proteins were expressed and produced in *E. coli* BL21(DE3)pLysS and purified for biochemical and structural studies. To evaluate nuclease activity and substrate specificity, various DNA and RNA substrates, including 5**′**- or 3**′**-FAM or biotin-labeled single- and double-stranded DNAs, stem-loop dsDNA or dsRNA with varying 5**′**- or 3**′**-overhang lengths, and poly(dA_12_), poly(dT_12_), and poly(dC_12_), were synthesized (Supplementary Table S1). Gh-TDH efficiently digested 5**′**-FAM-labeled stem-loop dsDNA in a concentration-dependent manner, producing a 3-nt 5**′**-FAM-labeled fragment through 3**′**–5**′** exonucleolysis (Fig. 1A, lanes 1–5 and 11–15). However, activity on 3**′**-FAM-labeled stem-loop substrates was significantly reduced, with cleavage of the FAM tag observed only at high enzyme concentrations (Fig. 1A, lanes 6–10 and 16–20). When incubated with 5**′**-FAM-labeled stem-loop dsDNA containing varying 3**′**-overhang lengths, Gh-TDH digested the overhangs to form blunt-ended dsDNA intermediates (Fig. 1B). These results indicate that Gh-TDH preferentially trims single-stranded 3**′**-overhangs in a 3**′**–5**′** exonucleolytic manner before processing dsDNA stem-loops. The 3**′**-FAM tag inhibits nuclease activity, reducing cleavage efficiency (Fig. 1A). Regardless of overhang length, Gh-TDH processes DNA until a blunt-ended dsDNA structure is formed (Fig. 1B), highlighting its substrate preference for 3**′**-overhangs. To further investigate the requirement for a free DNA terminus, we synthesized four linear ssDNA substrates with different terminal modifications, including 5**′**-FAM-labeled ssDNA, 5**′**-FAM-/3**′**-biotin-labeled ssDNA, 3**′**-FAM-labeled ssDNA, and 3**′**-FAM-/5**′**-biotin-labeled ssDNA (Supplementary Table S1, Entries 9, 11–13). Prior to the nuclease assay, all substrates were incubated with streptavidin. Streptavidin specifically binds to the biotin moiety on the biotinylated substrates, thereby generating sterically blocked DNA termini. The results showed that only the 5**′**-FAM-labeled ssDNA substrate was efficiently cleaved by Gh-TDH (Fig. 1C, lanes 1–5). In contrast, when the 3**′**-end was blocked by a biotin-streptavidin complex, Gh-TDH was unable to initiate degradation of the 5**′**-FAM-/3**′**-biotin-labeled substrate (Fig. 1C, lanes 6–10). Furthermore, following incubation with Gh-TDH, both the 3**′**-FAM-labeled substrate (Fig. 1D, lanes 1–5) and the 3**′**-FAM-/5**′**-biotin-labeled substrate (Fig. 1D, lanes 6–10) showed only trace amounts of released FAM signal, while no detectable DNA degradation from the 5**′** terminus was observed. Together, these findings provide strong evidence that Gh-TDH strictly requires a free 3**′** DNA terminus for substrate processing and functions as a 3**′**–5**′** exonuclease.

To further examine whether Gh-TDH is able to unwind dsDNA, a thermodynamically stable linear dsDNA substrate was used (Supplementary Fig. S1 and Supplementary Table S1, entries 9 and 10). Under the reaction conditions, Gh-TDH showed no detectable activity toward blunt-ended dsDNA, even at a high enzyme concentration of 20 μM. In contrast, the corresponding ssDNA with the same sequence was efficiently hydrolyzed by Gh-TDH (Supplementary Fig. S1). These results indicate that Gh-TDH functions as a 3**′**–5**′** exonuclease that preferentially degrades ssDNA and lacks the ability to unwind dsDNA prior to hydrolysis. In addition, we examined the nuclease activity of Gh-TDH toward a 66-mer circular ssDNA substrate and the corresponding linear ssDNA substrate containing the identical sequence (Supplementary Table S1, entries 14 and 15). Gh-TDH efficiently degraded the linear ssDNA substrate but exhibited no detectable activity toward the circular ssDNA substrate (Supplementary Fig. S2). These results further support the conclusion that Gh-TDH requires a free DNA end to initiate substrate processing.

To examine whether Gh-TDH is also capable of acting on RNA, we evaluated its ribonuclease activity using 5**′**-FAM-labeled stem-loop dsRNA with a 4-nt 3**′**-overhang (Supplementary Table S1, Entry 8). Gh-TDH was able to cleave RNA, although its efficiency was approximately 70% lower compared to its deoxyribonuclease activity on DNA (Supplementary Fig. S3). Substrate specificity studies using 5**′**-FAM-labeled poly(dA_12_), poly(dT_12_), and poly(dC_12_) DNA substrates showed that Gh-TDH efficiently degraded poly(dA_12_) and poly(dT_12_) in a time-dependent manner, with slightly reduced activity on poly(dC_12_). These results indicate that Gh-TDH removes nucleotides sequentially from the 3**′** end, leaving final 3-nt products, poly(dA_3_), poly(dT_3_), and poly(dC_3_) (Supplementary Fig. S4). We further performed kinetic analyses of Gh-TDH using poly(dA_12_), poly(dT_12_), and poly(dC_12_) as substrates. The results showed that Gh-TDH exhibited similar kinetic parameters toward poly(dA_12_) and poly(dT_12_), with *k*_cat_/*K*_m_ values of approximately 0.364 min⁻¹/2.745 μM and 0.357 min⁻¹/2.532 μM, respectively. In contrast, lower catalytic efficiency was observed for poly(dC_12_), with a *k*_cat_/*K*_m_ of approximately 0.276 min⁻¹/3.383 μM, indicating a modest preference for poly(dA_12_) and poly(dT_12_) over poly(dC_12_) (Supplementary Fig. S5). Both the nuclease activity assays and kinetic analyses indicate that Gh-TDH exhibits comparable activity toward poly(dA) and poly(dT), whereas its activity toward the poly(dC) substrate is reduced to approximately 60% of that observed for poly(dA).

The influence of divalent metal ions on Gh-TDH’s 3**′**–5**′** exonuclease activity was also investigated. Mg^2+^ and Co^2+^ significantly enhanced activity, Mn^2+^ exerted a marginal effect, while Ca^2+^, Ni^2+^, Cu^2+^, and Zn^2+^ showed no enhancement (Supplementary Fig. S6A and B). Analysis of Mg^2+^ concentrations showed that Gh-TDH exhibited optimal activity at 5–10 mM, while higher concentrations led to a dose-dependent decline (Supplementary Fig. S6C). In addition, ethylenediaminetetraacetic acid (EDTA) inhibited nuclease activity, confirming the requirement of divalent metal ions for Gh-TDH function (Supplementary Fig. S6C).

### Overall structure of Gh-TDH-DNA complexes

**Figure 2.**
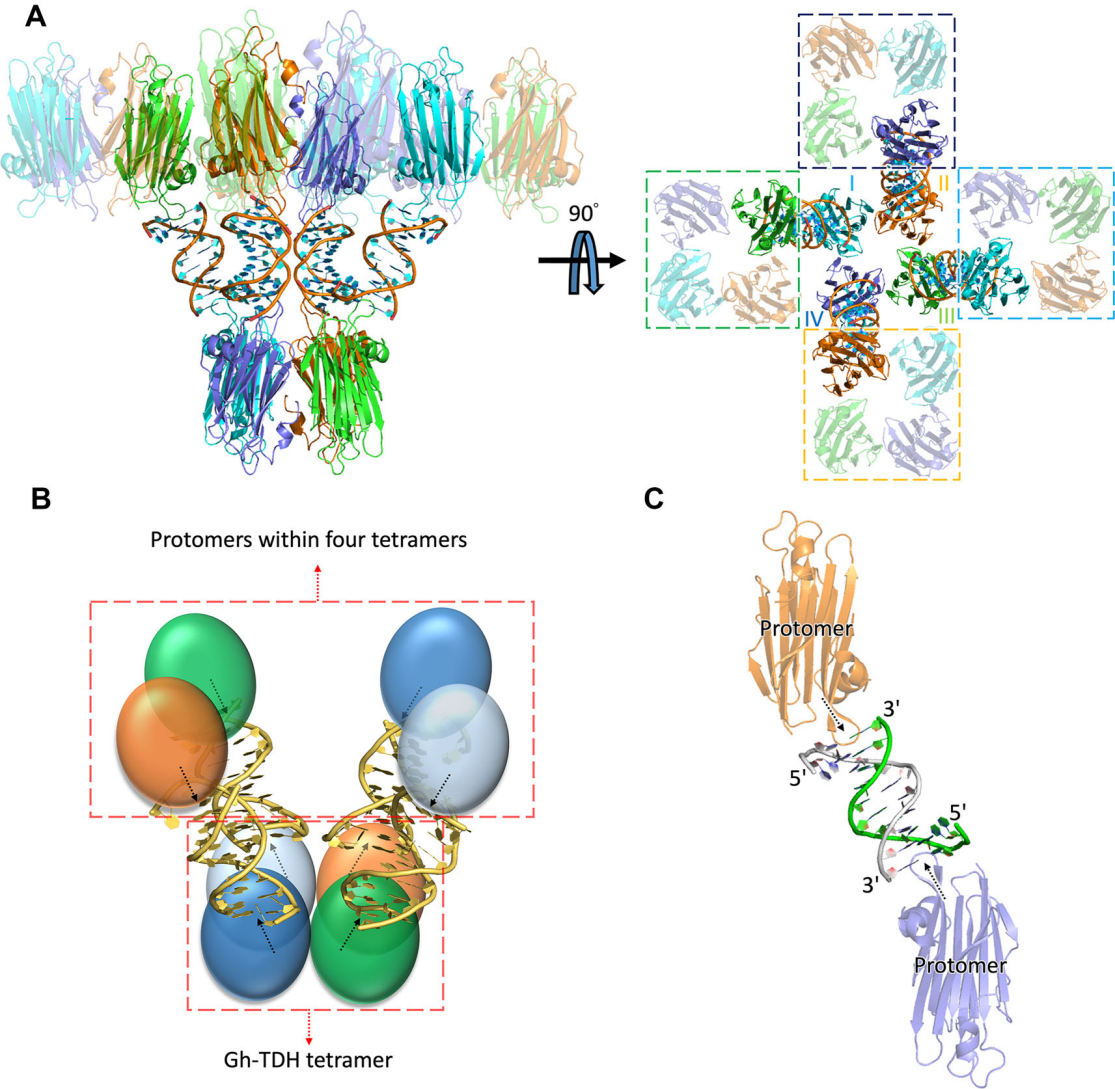
Crystal structures of Gh-TDH-DNA complex I. (**A**), Side (left) and top (right) views of complex I (right). The Gh-TDH tetramer is indicated by large dashed boxes in dark blue, cyan, yellow, and green. (**B**), Schematic model of the Gh-TDH-DNA complex I. Each color corresponds to a distinct protomer, and arrows indicate the direction of DNA binding. (**C**), Isolated protomer–DNA views of Gh-TDH–DNA complex I. Figure (**A**) shows only one assembly in which a Gh-TDH tetramer bound to DNA. The upper Gh-TDH tetramers are depicted in transparent representation. The crystal packing of Gh-TDH and the symmetry-expanded view are provided in Supplementary Fig. S8.

To investigate how Gh-TDH binds and processes DNA, we determined its crystal structure bound to a 12-nt ssDNA using molecular replacement with the Gh-TDH structure (PDB entry: 4WX3) [30]. Crystallization conditions, data collection, and refinement statistics are detailed in Supplementary Tables 2 and 3. The Gh-TDH-DNA complexes (complexes I and II), which display distinct *N*-terminal conformations, exhibit identical lattice packing and space group (P42_1_2), indicating that the crystals share the same symmetry and unit cell arrangement, which suggests that the differences in the observed structures are due to conformational variability rather than crystallization artifacts. In both complexes, eight ssDNA strands form four duplexes, each comprising 10 base-paired (C_3_–G_12_) nucleotides and a 2-nt unpaired 5**′**-overhang. One end of the duplexes binds the same Gh-TDH tetramer, while the other end associates with protomers from four different tetramers (Fig. 2). It is important to emphasize that TDH consistently forms a tetramer, regardless of the presence or absence of DNA binding, the type of DNA substrate, or the crystallization conditions. Complex I showed disordered *N*-terminal residues 1–11 (Supplementary Figs 7A and 8A), while in complex II, residues 7–11 are ordered and inserted into the tetramer’s central pore (Supplementary Figs 7B and 8B). Structure superposition reveals a conformational shift starting at Asp14 (D14), with a β-sheet stabilized by Gly62 (G62) and D14 in complex I replaced by a loop in complex II (Supplementary Fig. S7C). Despite *N*-terminal differences, the DNA conformation remains unchanged, suggesting *N*-terminal region variability does not affect DNA binding. Both complexes exhibited a narrowed central pore (19 vs. 20 Å in Gh-TDH) and a slight structural adjustment, with complex I showing a 5° rotation along the *x*-axis, resulting in a root mean square deviation (r.m.s.d.) of 1.54 Å relative to the Gh-TDH structure (Supplementary Fig. S9). These findings indicate minor conformational changes in the Gh-TDH tetramer accommodate binding of four duplexes.

Notably, previous studies have demonstrated that Gh-TDH exists as a tetramer in solution, consistent with its crystallographic structures [30]. In addition, transmission electron microscopy (TEM) analysis confirmed that TDH adopts a tetrameric state upon attachment to lipid membranes, forming a tetrameric pore structure [46]. Although the Gh-TDH-DNA complex structure shows that each TDH subunit can independently interact with DNA, the collective structural and biochemical evidence supports that Gh-TDH functions as a tetramer during DNA binding. Furthermore, the Gh-TDH-DNA complex structures reveal a crystal packing arrangement that could extend into a large polymeric assembly. To determine whether such an assembly occurs in solution, we performed dynamic light scattering (DLS) and native PAGE analysis using Gh-TDH in the absence of DNA and in the presence of varying concentrations of DNA. The results show that Gh-TDH remains predominantly in the tetrameric state under all tested conditions (Supplementary Fig. S10). These results suggest that the packing observed in the crystal structure likely reflects the mode of Gh-TDH–DNA binding interactions together with crystal packing effects, rather than a definitive physiological assembly. Furthermore, to determine the DNA-binding stoichiometry of Gh-TDH, we performed a Job plot analysis [44, 45]. The total molar amount of Gh-TDH (one polypeptide chain) and DNA substrate was held constant while the molar ratio of Gh-TDH to DNA was varied from 1:8 to 8:1, as shown in Supplementary Fig. S11. The result revealed that the highest level of product formation was observed when Gh-TDH and DNA were present at a molar ratio of 1:1. These results indicate that Gh-TDH binds and processes DNA with a 1:1 stoichiometry (*n* = 1), consistent with the Gh-TDH-DNA complex structures determined in this study, in which a single DNA molecule is bound to each Gh-TDH protomer.

**Figure 3.**
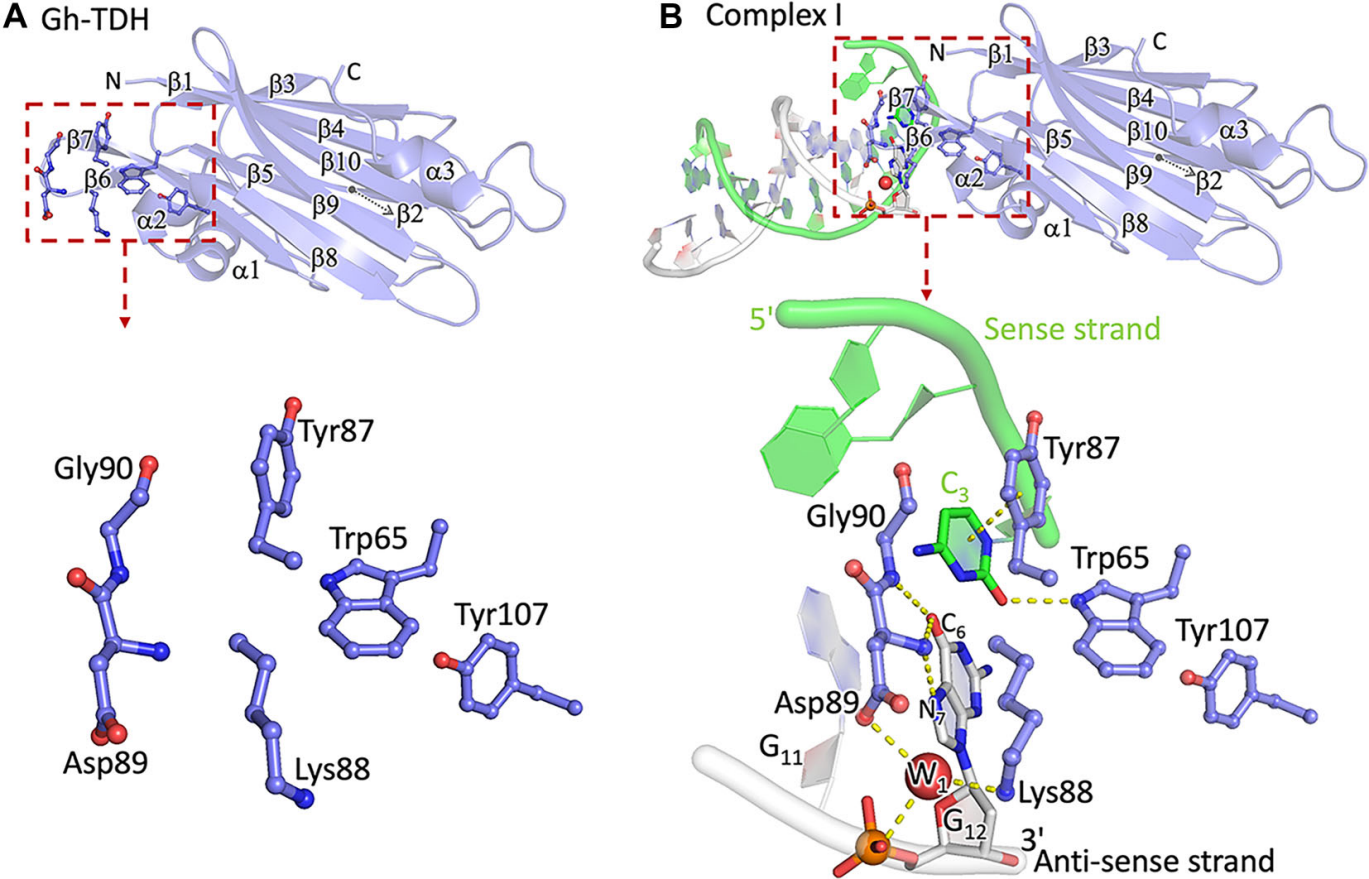
Structural analyses of the dsDNA-binding site in the Gh-TDH-DNA complex. (**A**), A protomer of tetrameric Gh-TDH is shown in blue, with the DNA-binding sites highlighted as balls and sticks. (**B**), A local view of protein–DNA interactions at the DNA terminal, where the sense strand is shown in green and the antisense strand in white. The residues involved in DNA binding are depicted in balls and sticks. The water molecule is shown as a red sphere (W_1_). Hydrogen bond interactions between the C_3_-G_12_ pair of the dsDNA and the β-hairpin (^87^YKDG^90^) of the protein are shown in yellow dashed lines.

The crystal structures of complexes I and II revealed that Tyr87 (Y87) of the Gh-TDH protomer caps the 5**′**-end of each DNA duplex (Fig. 3). In Gh-TDH, residues Y87–G90 form a β-hairpin structure between the sixth and seventh β-strands (Fig. 3A). Upon DNA binding, these residues undergo conformational changes to interact with the C_3_-G_12_ base pair (C_3_ on the sense strand and G_12_ on the anti-sense strand) (Fig. 3B and Supplementary Fig. S9B). Key interactions include the indole side chain of Trp65 (W65), which forms a 2.8 Å hydrogen bond with the carbonyl group of C_3_, and the phenolic ring of Y87, which engages in π-π stacking (3.9 Å) with C_3_’s aromatic pyrimidine ring. In the β-turn, the main chain amino group of Asp89 (D89) hydrogen bonds with the N7 amine (2.8 Å) and carbonyl group (3.4 Å) of G_12_, while the amino group of Gly90 (G90) forms a 3.0 Å hydrogen bond with G_12_’s carbonyl group. Additionally, the side chains of Lys88 (K88) and Asp89 (D89) create water-mediated hydrogen bonds with the phosphate backbone between G_11_ and G_12_. In complex I, interactions between K88, D89, and the 3**′**-end of the dsDNA stabilize their structural flexibility. This stabilization is reflected in the reduced B-factors for K88’s amino group in Gh-TDH compared to that in complex I (52.9–21.0). The elasticity of residues Y87–G90’s β-hairpin structure facilitates Gh-TDH’s capacity to adopt and bind diverse DNA structures effectively.

### Gh-TDH recognizes DNA substrates of varying lengths and sequences and mediates their cleavage

**Figure 4.**
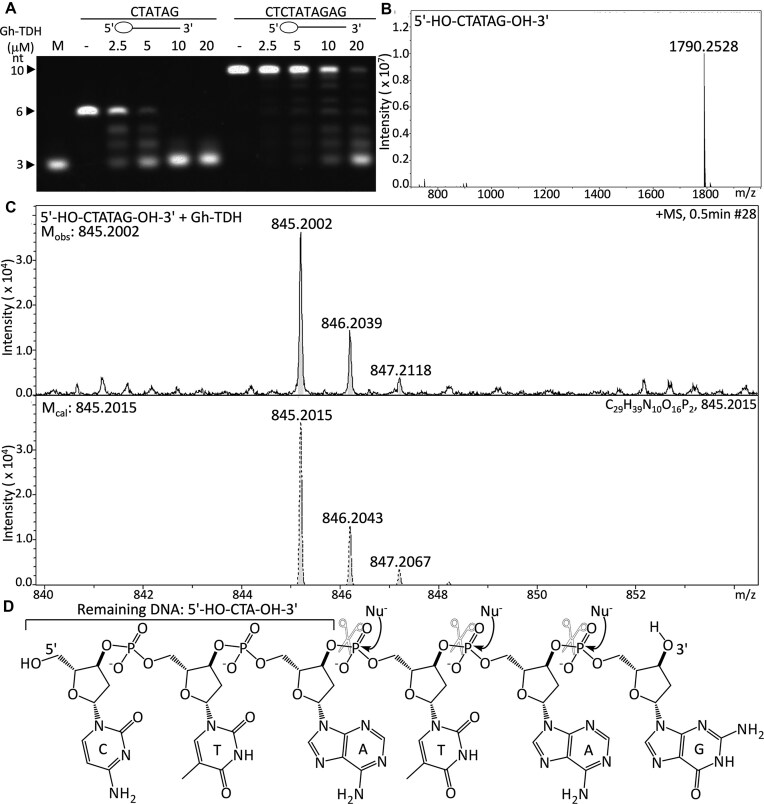
Nuclease activity assay of Gh-TDH against 6- and 10-nt DNA substrates. (**A**), Exonuclease activity of Gh-TDH digested 5′-FAM labeled 6-nt and 10-nt ssDNA. (**B**), HD Q-TOF spectrum of 6-nt 5′-OH-CTATAG-OH-3′ used for Gh-TDH nuclease activity assay. (**C**), HD Q-TOF spectrum of Gh-TDH-cleaved 6-nt 5′-OH-CTATAG-OH-3′. The singly charged ion 5′-OH-CTA-OH-3′ at m/z 845.2002 was observed in the Gh-TDH DNA cleavage product. (**D**), Chemical structure of 6-nt 5′-OH-CTATAG-OH-3′ and the site of Gh-TDH cleavage to 3-nt 5′-OH-CTA-OH-3′ shown in brackets.

To evaluate the impact of DNA sequence and length on Gh-TDH’s binding and cleavage activity, 10-nt and 6-nt DNA substrates (with or without 5**′**-FAM-labels) were used in nuclease assays and structural analysis (complexes III and IV). As shown in Fig. 4A, Gh-TDH cleaved both 10-nt and 6-nt DNAs in a concentration-dependent manner. Cleavage sites were identified through high-resolution mass analysis (HD Q-TOF) of a 6-nt DNA substrate with hydroxyl groups at both ends (Fig. 4B). The analysis revealed a molecular mass of 845.2002, matching the calculated mass of a CTA 3-mer with a 3**′**-hydroxyl group (Mw_calc._: 845.2015) (Fig. 4C), confirming that Gh-TDH cleaves the 3**′** O-P bond to produce a 3-mer DNA with a 3**′**-hydroxyl group (Fig. 4D).

**Figure 5.**
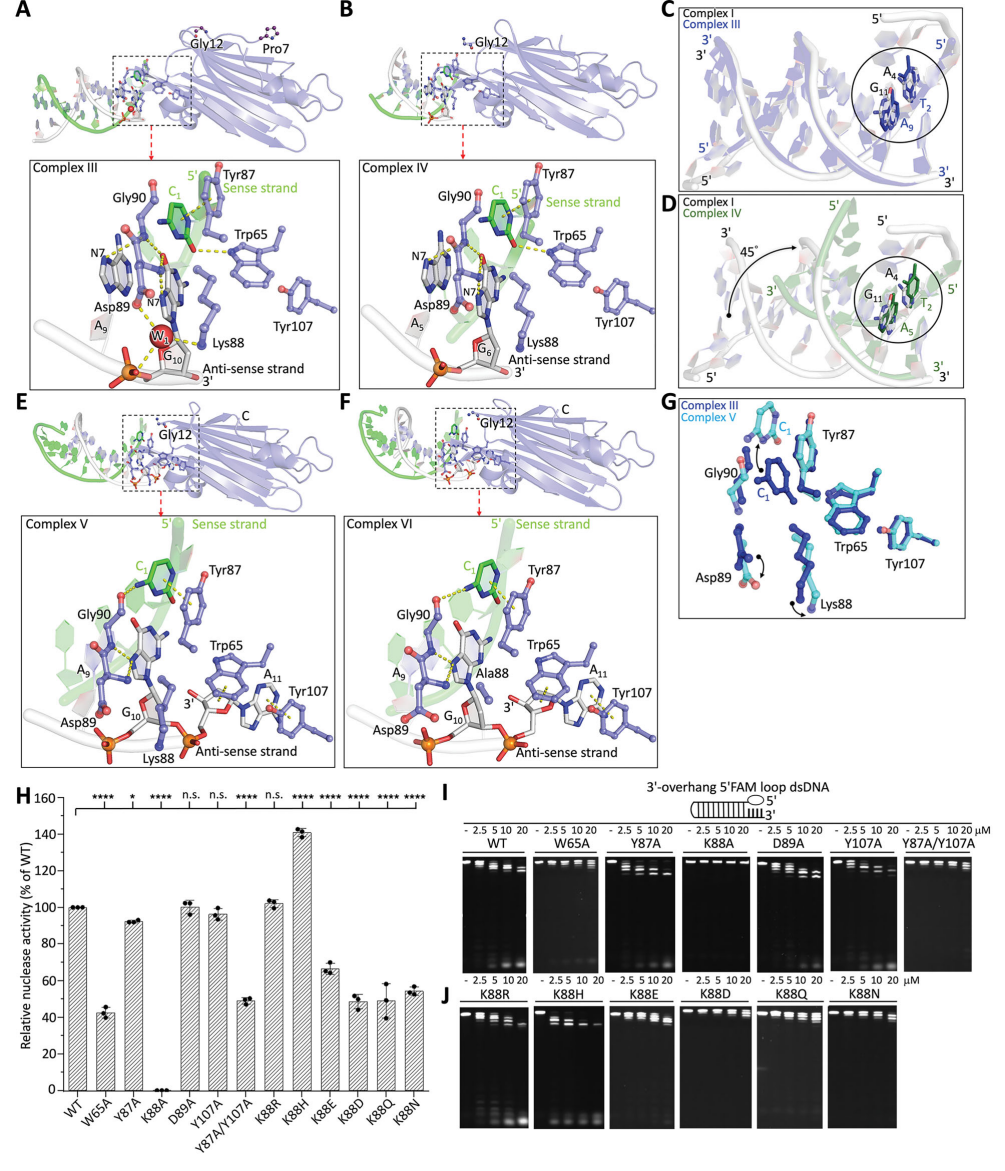
Binding of Gh-TDH to DNA ends with different sequences and mutagenesis analysis of Gh-TDH wild-type and its variants. (**A**), Complex III: crystal structure of Gh-TDH in complex with 10-nt dsDNA. (**B**), Complex IV: crystal structure of Gh-TDH in complex with 6-nt dsDNA. (**C**), Superposition of dsDNA in complex I (white cartoon) and complex III (blue cartoon). (**D**), Superposition of dsDNA in complex I (10 base pairs, white cartoon) and complex IV (6 base pairs, green cartoon), showing the 45° rotation of the helix due to variations in DNA length. (**E**), Complex V: crystal structure of Gh-TDH in complex with 11-nt dsDNA with 1-nt 3′-overhang. (**F**), Complex VI: crystal structure of Gh-TDH K88A variant in complex with 11-nt dsDNA with 1-nt 3′-overhang. (**G**), The structural difference between complex III (blue) and complex V (cyan). The movement is marked by three arrows, showing that the C_1_ base in complex V moves toward the hydroxyl group of Y87, accompanied by conformational changes in K88 and D89. The sense and antisense strands of the dsDNA are colored green and white, respectively. Hydrogen bonds are depicted as dashed lines, while key binding residues are shown as sticks. (**H**), Relative activities of Gh-TDH variants (W65A, Y87A, K88A, D89A, Y107A, Y87A/Y107A, K88R, K88H, K88E, K88D, K88Q, and K88N) were compared with Gh-TDH wild-type (WT) using a 5′-FAM-labeled stem-loop dsDNA with a 4-nt 3′-overhang. Activity was measured at 5 μM protein concentration and 0.5 μM substrate concentration. Data are shown as mean ± s.d. (*n* = 3). Statistical significance was determined using one-way ANOVA followed by Dunnett’s multiple comparisons test against WT (****p < 0.0001; ***p < 0.001; *p < 0.05; n.s., not significant). (**I**) and (**J**), 20% TBE urea polyacrylamide gel electrophoresis of DNA products after incubation with Gh-TDH WT or its variants.

We further determined Gh-TDH complex structures with 10-nt and 6-nt DNA, corresponding to complexes III and IV, respectively. Structural analysis revealed that the ssDNA used for co-crystallization was self-complementary and formed blunt-ended dsDNA (Fig. 5A and B and Supplementary Fig. S12). The *N*-terminal residues Pro7–Pro11, visible in complexes II and III, were absent in complex IV, where the *N*-terminus remained disordered, resembling complex I and the Gh-TDH structure (Fig. 5A and B). Despite differences in DNA length and sequence (A_9_ and A_5_ in complexes III and IV, vs. G_11_ in complex I) (Supplementary Table S2), the binding modes in complexes III and IV closely resemble that of complex I (Fig. 5A and B). In complex III, the indole side chain of W65 forms a hydrogen bond with C_1_’s carbonyl group (2.9 Å, sense strand), Y87’s phenolic side chain stacks with C_1_ (3.7 Å), and D89’s main chain amino group forms hydrogen bonds with G_10_’s (antisense strand) N7 amine (2.8 Å) and carbonyl group (3.5 Å). G90’s main chain amino group forms hydrogen bonds with A_9_’s N7 amine (3.5 Å) and G_10_’s carbonyl group (2.9 Å) in complex III (Fig. 5A), compared to a hydrogen bond with only the G_12_’s carbonyl group (2.9 Å) in complex I (Fig. 2B). Complex IV shows a nearly identical DNA-binding environment to complex III (Fig. 5B), with complexes III and IV displaying consistent interaction regions across the Gh-TDH-DNA complexes (Fig. 5C). In addition, variations in length result only in a shift of the DNA helix, as evidenced by the ∼45° rotation observed between complex I and complex IV (Fig. 5D). Together with the nuclease activity assays, these complex structures suggest that Gh-TDH can act on DNA substrates regardless of their length or sequence.

### Lys88 is an important residue for DNA cleavage

Alanine scanning mutagenesis of Gh-TDH residues near the DNA-binding site (W65, Y87, K88, D89, Y107A, and Y87/Y107) was performed to assess their role in nuclease activity using a 5**′**-FAM-labeled stem-loop dsDNA with a 4-nt 3**′**-overhang. The K88A variant showed the most significant reduction, completely losing exonuclease activity at 5 μM protein concentration (Fig. 5H and I). The crystal structure of the K88A variant and substrate-binding kinetics indicate that this mutation does not compromise protein folding or DNA-binding affinity (discussed further below). Mutations of Y87A, D89A, and Y107A had minimal impact, while W65A and Y87A/Y107A caused partial reductions.

Further testing of K88 mutants (K88E, K88D, K88Q, K88N, K88R, and K88H) showed that K88E, K88D, K88Q, and K88N reduced activity to 66%, 49%, 49%, and 54%, respectively (Fig. 5H and J), while K88R retained wild-type activity and K88H showed a slight increase. The involvement of lysine as a key residue in nuclease catalysis has been reported previously; for example, K131 in bacteriophage lambda exonuclease has been proposed to activate the nucleophilic water molecule or stabilize a hydroxide ion during catalysis [47]. Our results suggest that K88 in Gh-TDH may play an analogous role to that in bacteriophage lambda exonuclease, potentially contributing to stabilization of the catalytic state or functioning as a general base.

To further clarify whether K88 primarily contributes to catalysis or substrate binding, we examined the DNA-binding affinity of the K88A variant using bio-layer interferometry (BLI). The dissociation constants (*K*_D_) for Gh-TDH wild-type and the K88A variant toward stem-loop dsDNA with a 4-nt 3′-overhang were measured to be 14.8 and 22.4 μM, respectively. Furthermore, the *K*_D_ values toward ssDNA substrates, including poly(dA), poly(dT), and poly(dC), were measured to be 2.39–3.96 μM for the wild-type protein and 3.64–5.01 μM for the K88A variant (Supplementary Figs. 13 and 14). These results indicate only a slight reduction in DNA-binding affinity for the mutant. Structural analysis of the Gh-TDH-DNA complex (Fig. 3B) reveals that residues K88 and D89 engage DNA through a water-mediated interaction. Replacement of K88 with alanine may alter this water network and contribute to the modest change in *K*_D_. Overall, the DNA-binding affinity of the K88A variant remains similar to that of the wild type, indicating that the loss of exonuclease activity cannot primarily be attributed to impaired DNA binding (Fig. 5H and I), but rather suggests a direct role of K88 in catalyzing DNA hydrolysis. In addition, these results suggest that Gh-TDH exhibits a moderate difference in binding affinity toward different DNA substrates, including poly(dA), poly(dT), poly(dC), and the stem-loop DNA.

To further investigate the effect of the K88 mutation on DNA binding and trimming, Gh-TDH wild-type and the K88A variant were co-crystallized with an 11-nt ssDNA, forming complex V and complex VI, respectively (Supplementary Tables 2 and 3). In solution, the two ssDNA strands form a dsDNA with a 1-nt 3**′**-overhang and a 10-nt duplex. In both complexes, the first 11 *N*-terminal residues are disordered, as seen in Gh-TDH and complex I (Fig. 5E and F and Supplementary Fig. S15). Crystal structures revealed that A_11_ of the antisense strand forms sugar–π and π–π interactions with W65 and Y107, respectively, differing from the C_3_/G_12_, C_1_/G_10_, and C_1_/G_6_ base pairs observed in complexes I–IV (Figs 3B and 5). In addition, Y87 still forms π-π stacking with C_1_, but C_1_ shifts closer to the hydroxyl group of Y87 (Fig. 5G). Together with BLI binding analysis, the structure of the K88A in complex with DNA substrate further demonstrates that mutation at K88 does not perturb the overall enzyme structure nor affect DNA substrate binding.

Intriguingly, in the crystal structure of complex V, two distinct DNA-binding modes were observed at the two ends of the DNA. These may arise from conformational flexibility and dynamics, leading to subtle differences in DNA recognition. One mode resembles that of complexes I–IV, while the other is similar to complex VI, with the key distinction being that the binding residue in C_1_ shifts from Gly90 to Trp65 (Supplementary Fig. S15A). In complexes V and VI, D89 forms a hydrogen bond with the N7 amine of G_10_ (the penultimate base), similar to its interaction with the final G base in complexes I–IV. However, in complex VI, G90 forms a hydrogen bond with the N7 amine of G_10_ (the penultimate G), unlike in complexes I–IV, where G90 forms a hydrogen bond with the carbonyl group of the final G (G_12_ in complexes I and II, G_10_ in complex III, G_6_ in complex IV) (Figs 3B and 5). Additionally, G90’s carbonyl group forms a hydrogen bond with the C-4 amino group of C_1_ (sense strand) in complex VI, a bond absent in complexes I–IV. Water molecules bound to K88 and D89 in complexes I–III are not observed in complexes IV–VI (Supplementary Fig. S16A–F). Structural analysis of complexes V and VI, along with BLI results, supports that the K88A variant does not affect Gh-TDH’s binding affinity or mode, reinforcing K88’s role as a catalytic residue. The differences in binding patterns between complexes III and V suggest that Gh-TDH adapts to various DNA structures to facilitate cleavage (Fig. 5G).

## Discussion

This study is the first to identify Gh-TDH, a hemolysin in *Vibrio* species, with nuclease activity, revealing its distinct structural basis compared to other known exonucleases. Based on structural comparison using the Dali server, several proteins with folds similar to Gh-TDH were identified. Although the sequence identity is low (up to ∼12%), many of these proteins adopt a β-sandwich architecture similar to that of Gh-TDH (Supplementary Fig. S17). Seven of the closest structural homologs belong to the pore-forming toxin family, in addition to one lectin [48–55]. However, despite the apparent structural similarity revealed by the Dali search, none of these proteins have been reported to interact with DNA or to possess nuclease activity. In addition, Gh-TDH efficiently processes both ssDNA and stem-loop dsDNA with 3**′**-overhang substrates; it shows no significant sequence or structural similarity to known nucleases (Supplementary Fig. S18). In contrast to well-characterized 3**′**–5**′** exonucleases or other nucleases, including members of the DEDD and DEK superfamilies, which typically use conserved aspartate (Asp) or glutamate (Glu) residues to coordinate divalent metal ions and stabilize the transition state during phosphodiester bond cleavage [38, 56–59], Gh-TDH lacks these canonical nuclease signature motifs and functional residues. Structural analysis shows that members of the DEDD superfamily, such as ExoX and TREX2, utilize a loop between β1 and β2 strands or an α-helical loop structure to specifically bind to the non-scissile strand of dsDNA [60, 61]. Similarly, the complex structure of Gh-TDH reveals that the enzyme primarily relies on aromatic residues Trp65 and Tyr87 to interact with the non-scissile strand. Additionally, while the general base in DEDD family enzymes is typically located within a loop (e.g. TREX2’s α6–α7 loop) [38], in Gh-TDH, the critical residue Lys88 is located within a unique β-turn (K88 to G90) which plays a critical role in DNA binding and cleavage.

Most nucleases rely on divalent metal ions to facilitate catalysis [38]. Our study shows that Gh-TDH functions as a metal ion-dependent nuclease despite lacking the canonical conserved motif of 3**′**–5**′** exonucleases. Mutational analysis of acidic residues located near the DNA-binding site (D89, D117, and E118) revealed no significant impact on nuclease activity (Supplementary Fig. S16G), indicating that these residues are not essential catalytic ligands and may, at most, play auxiliary roles in metal ion coordination. Notably, previous studies have shown that in many bimetallic nucleases, divalent metal ions primarily interact with the phosphate groups of the substrate or are coordinated via water molecules, rather than being tightly coordinated by enzyme residues [38, 62, 63]. Although we were unable to unambiguously define the position of the Mg^2+^ in our complex structures, mutagenesis analysis suggests that the metal ions may be primarily coordinated by the phosphate groups of the DNA or indirectly through water-mediated interactions.

In most nucleases, a histidine or tyrosine residue positioned near the active site functions as a general base to activate a water molecule for nucleophilic attack during DNA cleavage [38]. However, mutations of Y87 and Y107 in Gh-TDH had little effect on nuclease activity (Fig. 5H). In contrast, mutations at K88 significantly affected activity, highlighting its functional importance. Previous studies on bacteriophage lambda exonuclease have shown that a lysine residue K131 can serve as a general base or stabilize a hydroxide ion to promote DNA hydrolysis [47]. The Gh-TDH structure reveals that K88 interacts with the DNA 3**′**-end through a water-mediated network involving water molecules W_2_, W_3_, and W_4_ (Supplementary Fig. S16A–C). Together with mutagenesis and structural analysis, these observations suggest that K88 in Gh-TDH likely functions in a manner analogous to lambda exonuclease K131, acting as a general base to facilitate DNA cleavage. These findings indicate that TDH represents a previously uncharacterized class of nuclease, distinct from the canonical DEDD- and DEK-motif nuclease superfamilies [47, 61].

Sequence alignment between TDH and these representative nucleases reveals no significant sequence homology, indicating that TDH does not share sequence conservation with either family (Supplementary Fig. S18A). Likewise, NM23-H1 (also known as NME1) was originally identified as a nucleoside-diphosphate kinase, but was later discovered to be bi-functional, both a kinase and a 3**′**–5**′** exonuclease [64]. Similar to TDH, NME1 mediates DNA exonucleolytic degradation despite lacking the conserved catalytic residues typical of DEDD 3**′**–5**′** exonucleases. Structural comparison further highlights their divergence, whereas TREX2 and bacteriophage lambda exonuclease primarily adopt α-helical structures, TDH exhibits a β-sheet-rich fold (Supplementary Fig. S18B). Therefore, unlike these conventional nucleases, TDH lacks conserved nuclease motifs and exhibits a distinct amino acid sequence, structural fold, and DNA-binding mode.

**Figure 6.**
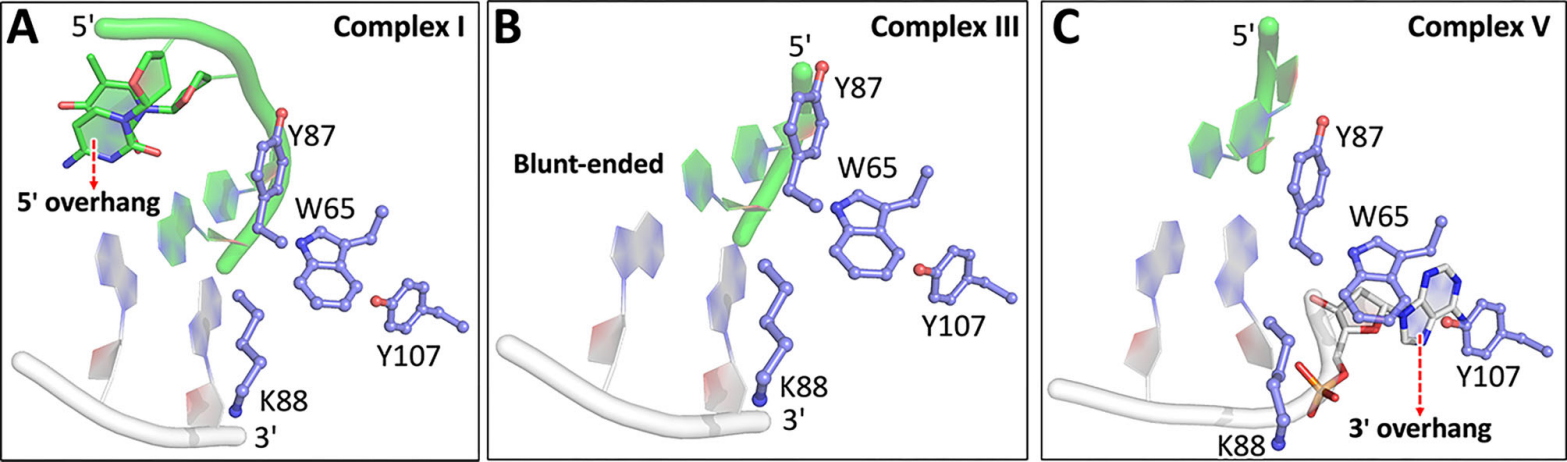
Structural comparison of Gh-TDH in complex with distinct DNA substrates. (**A**), Close-up view of 2-nt 5′-overhang dsDNA bound in complex I. (**B**), Close-up view of blunt-ended 10-nt dsDNA bound in complex III. (**C**), Close-up view of 1-nt 3′-overhang dsDNA bound in complex V. Residues involved in DNA binding are depicted in balls and sticks.

Comparison of the Gh-TDH complex structures with 5**′**-overhang, blunt-ended, and 3**′**-overhang dsDNA substrates (complexes I, III, and V) shows that all dsDNA substrates are recognized through a similar set of binding residues. However, in complex I, the 5**′**-overhang dsDNA does not form direct interactions with the protein and is not positioned near the proposed K88 catalytic site (Fig. 6A), consistent with its lack of preferential hydrolysis. Comparison of the blunt-ended and 3**′**-overhang dsDNA complexes reveals that the additional 3**′**-overhang nucleotides extend toward the active site and are stabilized by interactions with W65 and Y107 through π–π interactions and hydrogen bonds (Fig. 6B and C). Notably, the phosphodiester bond predicted to undergo cleavage is positioned adjacent to the proposed K88 catalytic site (Fig. 6C). These structural observations support that Gh-TDH functions as a 3**′**–5**′** exonuclease.

In addition, structural comparison of complexes I–VI revealed only minor conformational changes in the hydrophobic pocket residues Y87, W65, and Y107, despite variations in DNA binding. A consistent π–π stacking interaction between Y87 and the first cytosine (sense strand) was observed across all complexes, with slight conformational shifts in complexes V and VI (Fig. 5G). In complexes I–IV, W65 forms a hydrogen bond with the first cytosine (sense strand), while in complexes V and VI, a sugar–π interaction with A_11_ (antisense strand) is observed (Fig. 5). Y107 interacts minimally with DNA in complexes I–IV, but forms a π–π interaction with A_11_ (antisense strand) in complexes V and VI. These shifts likely enable Gh-TDH to distinguish between scissile and non-scissile strands, facilitating precise cleavage at the 3**′**-end of the ssDNA substrate (Fig. 5G). The reduced exonuclease activity of the W65A and Y87A/Y107A variants further highlights the importance of these residues in efficient DNA cleavage (Fig. 5H). Notably, our previous studies on Gh-TDH membrane binding identified residues W65 and Y87 as important contributors to membrane association [30], suggesting that these two residues are involved in both Gh-TDH membrane binding and DNA binding.

In addition to *G. hollisae*, several *Vibrio* species also carry the *tdh* genes. Sequence alignment of Gh-TDH with selected TDH homologs from other species (Supplementary Fig. S19) revealed that Lys88, which is essential for nuclease activity, is highly conserved across TDH homologs from different *Vibrio* species. Moreover, the key DNA-binding residues in Gh-TDH, Trp65, Tyr87, Gly90, and Tyr107, are also conserved, with the exception of position 107, which is replaced by Phe in *V. harveyi* TDH. We further performed nuclease activity assays on Vp-TDH, confirming that it also exhibits nuclease activity (Supplementary Fig. S20). These results suggest that TDHs with high sequence similarity may generally share this enzymatic function, indicating that the nuclease activity observed in this family of pore-forming toxins is not a coincidental property but an intrinsic functional feature of TDHs.

The discovery of Gh-TDH’s intrinsic nuclease activity provides new insight into its role as a multifunctional virulence factor. While Gh-TDH has been extensively characterized for its hemolytic, membrane-binding, cytotoxic, cardiotoxic, and hepatotoxic properties, the identification of nuclease activity broadens its pathogenic capabilities. Based on our findings, the excised nucleotides are unlikely to be reutilized by Gh-TDH, as it lacks polymerase or DNA repair-associated domains [38]. Rather than cooperating with host DNA replication machinery, Gh-TDH likely promotes genomic instability by cleaving host DNA. Consistent with previous studies, TDH has been shown to induce mitochondrial damage and trigger several hallmark features of apoptosis-like programmed cell death [18, 65]. We therefore propose that Gh-TDH’s exonuclease activity represents a virulence mechanism distinct from canonical proofreading exonucleases, specifically targeting host genome integrity. Additionally, the degradation of host DNA may supply nutrient sources, such as nitrogen and phosphate, supporting bacterial survival and colonization in nutrient-limited environments like damaged tissues. Importantly, this nuclease function may act synergistically with Gh-TDH’s hemolytic and apoptotic activities, amplifying host cell damage and promoting bacterial dissemination. Together, these findings suggest that Gh-TDH functions not only as a pore-forming toxin but also as a strategic effector that enables *G. hollisae* to exploit host resources and persist in both host-associated and environmental niches. Although the physiological role of TDH’s nuclease activity *in vivo* remains unclear, this study is the first to demonstrate that a hemolysin possesses nuclease activity, providing novel insights into its multifaceted mechanisms of virulence and laying the groundwork for further investigations.

## Supplementary Material

gkag679_Supplemental_File

## Data Availability

The data associated with this study are available within the article and Supplementary Information. All the coordinates and structure factors have been deposited in the Protein Data Bank with DOIs https://doi.org/10.2210/pdb9lcs/pdb, https://doi.org/10.2210/pdb9l8e/pdb, https://doi.org/10.2210/pdb9l9m/pdb, https://doi.org/10.2210/pdb9lcm/pdb, https://doi.org/10.2210/pdb9lcu/pdb, and https://doi.org/10.2210/pdb9lcw/pdb. Source data are provided with this paper. All other data are available from the corresponding author on request.
