# Supplementary material for: Structural and biochemical characterization of Grimontia hollisae thermostable direct hemolysin with DNA reveals first Vibrio hemolysin with nuclease activity
Source: Nucleic Acids Res. 2026 Jul 6;54(13):gkag679. doi: 10.1093/nar/gkag679 (PMC13335484; doi:10.1093/nar/gkag679)
Supplement: gkag679_Supplemental_File [file gkag679_supplemental_file.pdf]

Supplementary Information for

Structural and Biochemical Characterization of *Grimontia hollisae* Thermostable Direct Hemolysin with DNA Reveals First *Vibrio* Hemolysin with Nuclease Activity

Po-Yun Hsiao[1], Yu-Kuo Wang[1], Sheng-Cih Huang[1], Feng-Pai Chou[1], Tzu-Yu Huang[1], Yen-Cheng Lin[1], You-Min Kuo[1], Tung-Kung Wu[1,2,3,*], and Chin-Yuan Chang[1,2,3,4,*]

[1]Department of Biological Science and Technology, National Yang Ming Chiao Tung University, Hsinchu 30010, Taiwan, Republic of China
[2]Center for Emergent Functional Matter Science, National Yang Ming Chiao Tung University, Hsinchu 30010, Taiwan, Republic of China
[3]Center for Intelligent Drug Systems and Smart Biodevices (IDS2B), National Yang Ming Chiao Tung University, Hsinchu 30010, Taiwan, Republic of China
[4]Department of Biomedical Science and Environmental Biology, Drug Development and Value Creation Research Center, Kaohsiung Medical University, Kaohsiung 807, Taiwan, Republic of China

Table S1. Nucleic acid substrates used for nuclease activity, LC-ESI-MS analysis and Bio-Layer Interferometry (BLI).

Entry	Substrate name	DNA sequence
1	5′-FAM Stem-loop DNA with 4-nt 3′-overhang	5′- F-CTCTATAGAG**TTTT**CTCTATAGAG**TTCT** -3′
2	3′-FAM Stem-loop DNA with 4-nt 3′-overhang	5′- CTCTATAGAG**TTTT**CTCTATAGAG**TTCT**-F -3′
3	5′-FAM Stem-loop DNA with 4-nt 5′-overhang	5′- F-**TTCT**CTCTATAGAG**TTTT**CTCTATAGAG -3′
4	3′-FAM Stem-loop DNA with 4-nt 5′-overhang	5′- **TTCT**CTCTATAGAG**TTTT**CTCTATAGAG-F -3′
5	5′-FAM Stem-loop DNA (blunt-ended)	5′- F-CTCTATAGAG**TTTT**CTCTATAGAG -3′
6	5′-FAM Stem-loop DNA with 2-nt 3′-overhang	5′- F-CTCTATAGAG**TTTT**CTCTATAGAG**TT** -3′
7	5′-FAM Stem-loop DNA with 8-nt 3′-overhang	5′- F-CTCTATAGAG**TTTT**CTCTATAGAG**TTCTTTCT** -3′
8	5′-FAM Stem-loop RNA with 4-nt 3′-overhang	5′- F-CUCUAUAGAG**UUUU**CUCUAUAGAG**UUCU** -3′
9	5′-FAM ssDNA 20-nt	5′- F-ACTGGACAAATACTCCGAGG -3′
10	5′-FAM dsDNA 20-nt	5′- F-ACTGGACAAATACTCCGAGG -3′<br>3′- TGACCTGTTTATGAGGCTCC -5′
11	5′-FAM 3′-biotin ssDNA 20-nt	5′- F-ACTGGACAAATACTCCGAGG-B -3′
12	3′-FAM ssDNA 20-nt	5′- ACTGGACAAATACTCCGAGG-F -3′
13	3′-FAM 5′-biotin ssDNA 20-nt	5′- B-ACTGGACAAATACTCCGAGG-F -3′
14	Circular ssDNA 66-nt	5′- TCAGTGTTTTTTTCGTCGATTGCAGTAACTCCC<br>CACAACCTCTTTTTCGATGCTTTTTTTGTGCGA -3′
15	Linear ssDNA 66-nt	5′- TCAGTGTTTTTTTCGTCGATTGCAGTAACTCCC<br>CACAACCTCTTTTTCGATGCTTTTTTTGTGCGA -3′
16	5′-FAM poly(dA_{12})	5′- F-AAAAAAAAAAAA -3′
17	5′-FAM poly(dT_{12})	5′- F-TTTTTTTTTTTT -3′
18	5′-FAM poly(dC_{12})	5′- F-CCCCCCCCCCCC -3′
19	5′-FAM DNA 10-nt	5′- F-CTCTATAGAG -3′
20	5′-FAM DNA 6-nt	5′- F-CTATAG -3′
21	DNA 6-nt	5′- CTATAG -3′
22	5′-biotin Stem-loop DNA with 4-nt 3′-overhang	5′- B-CTCTATAGAG**TTTT**CTCTATAGAG**TTCT** -3′
23	5′-biotin poly(dA_{12})	5′- B-AAAAAAAAAAAA -3′
24	5′-biotin poly(dT_{12})	5′- B-TTTTTTTTTTTT -3′
25	5′-biotin poly(dC_{12})	5′- B-CCCCCCCCCCCC -3′

The underline regions are the paired region of DNA substrates or RNA substrates
Bold-black: loop
Bold-red: overhang
F: FAM [5-Carboxyfluorescein]
B: biotin

Table S2. Crystallization conditions of different Gh-TDH-DNA complexes.

**2-nt long 5'-overhang dsDNA (complex I)**	**6-nt blunt-ended dsDNA (complex IV)**
Protein: wild-type Gh-TDH<br>Input DNA: CTCACTATAGGG<br>Condition for crystallization:<br>0.2 M Lithium sulfate monohydrate, 0.1 M Sodium citrate tribasic dihydrate pH 5.0, 26% v/v Polyethylene glycol 200	Protein: wild-type Gh-TDH<br>Input DNA: CTATAG<br>Condition for crystallization:<br>30% (v/v) MPD 100 mM Sodium acetate/ Hydrochloric acid pH 4.6 20 mM Calcium chloride
**2-nt long 5'-overhang dsDNA with N-ter (complex II)**	**1-nt long 3'-overhang dsDNA (complex V)**
Protein: wild-type Gh-TDH<br>Input DNA: CTCACTATAGGG<br>Condition for crystallization:<br>0.2 M Lithium sulfate monohydrate, 0.1 M BIS-TRIS pH 5.5, 25% v/v Polyethylene glycol 3,350	Protein: wild-type Gh-TDH<br>Input DNA: CTCTATAGAGA<br>Condition for crystallization:<br>200 mM Ammonium sulfate, 100 mM Bis Tris/ Hydrochloric acid pH 5.5
**10-nt blunt-ended dsDNA with N-ter (complex III)**	**1-nt long 3'-overhang dsDNA(complex VI)**
Protein: wild-type Gh-TDH<br>Input DNA: CTCTATAGAG<br>Condition for crystallization:<br>0.2 M Lithium sulfate monohydrate, 0.1 M BIS-TRIS pH 5.5, 25% v/v Polyethylene glycol 3,350	Protein: K88A Gh-TDH<br>Input DNA: CTCTATAGAGA<br>Condition for crystallization:<br>200 mM Ammonium sulfate, 100 mM Bis Tris/ Hydrochloric acid pH 5.5

Underline: paired region of DNA substrates

Table S3. Crystallographic data and refinement statistics.

	Complex I	Complex II	Complex III	Complex IV	Complex V	Complex VI
**Data collection**						
Space group	$P42_12$	$P42_12$	$P42_12$	$C222_1$	$P2_1$	$P2_1$
Cell dimensions						
a, *b*, *c* (Å)	79.65, 79.65, 74.24	79.71, 79.71, 74.85	79.73, 79.73, 75.43	112.84, 122.56, 60.84	79.79, 61.64, 113.46	81.60, 61.66, 113.28
α, β, γ (°)	90.00, 90.00, 90.00	90.00, 90.00, 90.00	90.00, 90.00, 90.00	90.00, 90.00, 90.00	90.00, 104.67, 90.00	90.00, 106.62, 90.00
Resolution (Å)	25.70–1.58 (1.61–1.58)	25.22–1.37 (1.42–1.37)	25.07–1.42(1.47–1.42)	27.67–1.82 (1.85–1.82)	28.23–2.32 (2.40–2.32)	29.78–2.71 (2.74–2.69)
R_{sym} or R_{merge}	6.8 (82.5)	4.5 (48.7)	5.8 (58.8)	7.2 (47.2)	5.2 (34.1)	8.8 (43.3)
$I / \sigma I$	41.3 (3.8)	37.5 (4.0)	31.7 (2.8)	26.0 (3.6)	20.4 (3.0)	11.0 (2.1)
Completeness (%)	100.0 (100)	100.0 (100.0)	99.7 (100.0)	99.9 (98.6)	99.2 (99.3)	85.1 (98.0)
Redundancy	12.4 (12.5)	8.9 (8.9)	7.9 (7.9)	7.3 (6.6)	3.3 (3.1)	2.7 (3.0)
**Refinement**						
Resolution (Å)	25.70–1.58	25.22–1.37	25.07–1.42	27.67–1.82	28.23–2.32	29.78–2.71
No. reflections	31868	47798	43011	33650	41292	20729
R_{work} / R_{free}	0.185/0.197	0.186/0.199	0.185/0.205	0.165/0.195	0.215/0.251	0.248/0.317
No. atoms						
Protein	1241	1300	1252	2467	4810	4713
DNA	243	243	202	119	446	446
Water	148	212	174	218	187	62
***B*-factors**						
Protein	14.4	15.3	15.9	21.9	33.3	46.7
DNA	45.6	35.9	40.8	50.8	77.2	86.3
Water	25.5	28.2	26.3	31.0	34.8	38.2
R.m.s. deviations						
Bond lengths (Å)	0.0035	0.0036	0.0047	0.0093	0.0073	0.0049
Bond angles (°)	1.15	1.18	1.20	1.47	1.46	1.15

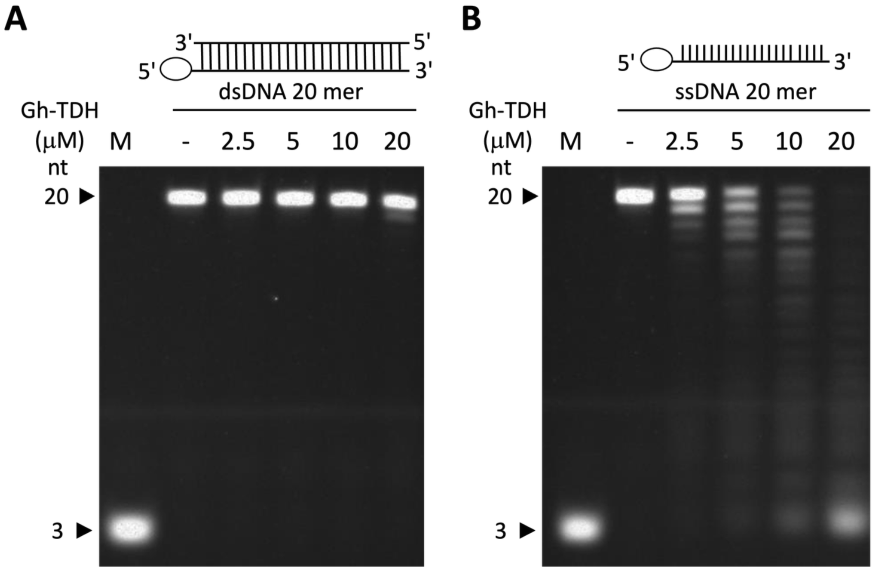

Fig. S1. Nuclease activity assay to evaluate the dsDNA unwinding capability of Gh-TDH. (A) Gh-TDH was incubated with blunt-ended dsDNA labeled with 5'-FAM at one terminus. (B) Gh-TDH was incubated with the corresponding 5'-FAM-labeled single-stranded DNA of the same sequence.

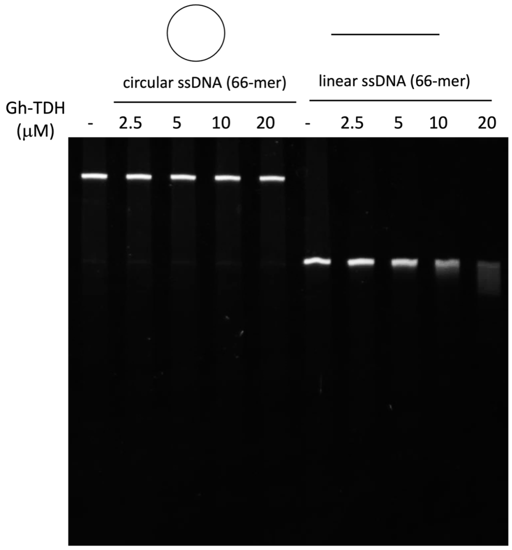

Fig. S2. Nuclease activity assay to evaluate the cleavage of circular ssDNA and linear ssDNA by Gh-TDH. Gh-TDH was incubated with circular ssDNA (66-mer) and the corresponding linear ssDNA of the same sequence. DNA products were visualized directly using a DNA-staining dye (SYBR Gold).

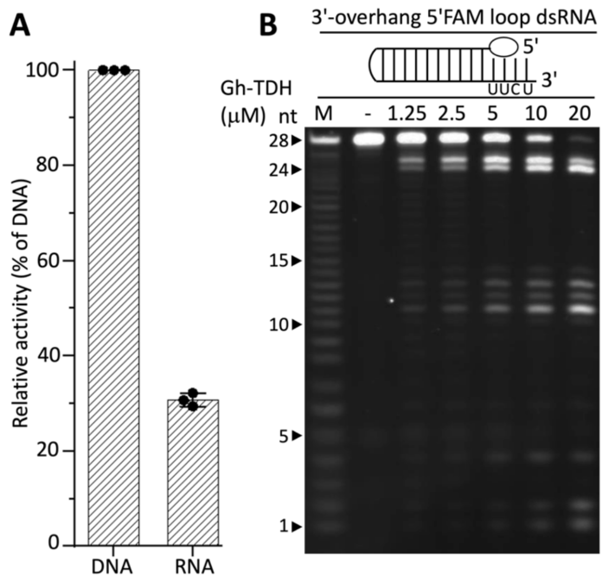

Fig. S3. Ribonuclease activity of Gh-TDH. (A) Comparison of Gh-TDH nuclease activity on DNA and RNA. Specific activity against DNA (Fig. 1A, lane 3) and RNA was measured with 5 μM Gh-TDH. Mean ± SD (n = 3) was shown. (B) Serial dilutions of Gh-TDH (1.25, 2.5, 5, 10, and 20 μM) were incubated with 0.5 μM FAM-labeled stem-loop dsRNA substrate. Lane M represents RNA treated with NaOH, used as a control marker.

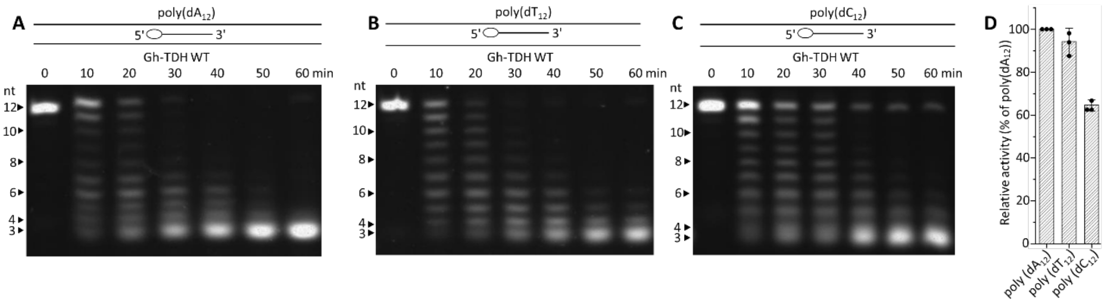

Fig. S4. Characterization of the base preference of Gh-TDH nuclease activity. (A) Nuclease activity of Gh-TDH digested poly(dA_{12}). (B) Nuclease activity of Gh-TDH digested poly(dT_{12}). (C) Nuclease activity of Gh-TDH digested poly(dC_{12}). 10 μM Gh-TDH and poly(dN_{12}) were incubated at 37 °C for 60 min, with gel samples taken every 10 min. All ssDNA was cleaved by Gh-TDH until 3 nucleotides remain. (D) Relative nuclease activity of Gh-TDH digested poly(dA_{12}), poly(dT_{12}), and poly(dC_{12}). The relative activities were determined based on the reaction at the 10 min. Mean ± SD (n = 3) was shown.

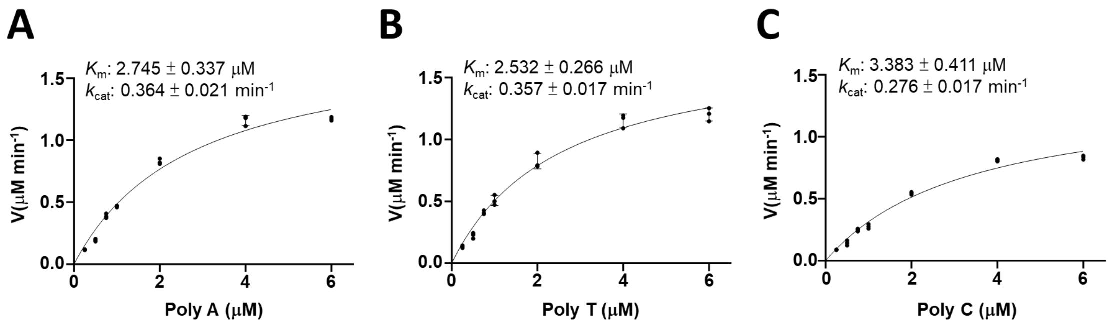

Fig. S5. The Michaelis-Menten kinetics of Gh-TDH. The Gh-TDH reaction against series concentration of (A) poly(dA_{12}), (B) poly(dT_{12}), and (C) poly(dC_{12}). Mean ± SD (n = 3) was shown.

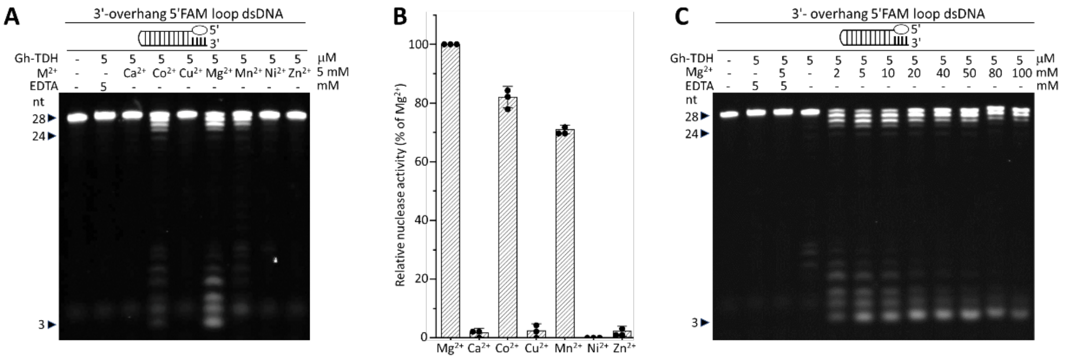

Fig. S6. Effect of divalent metal ions on Gh-TDH 3'–5' nuclease activity. (A) Effect of different divalent metal ions on Gh-TDH nuclease activity. (B) Relative nuclease activity of Gh-TDH in the presence of various divalent metal ions. Mean ± SD (n = 3) was shown. (C) Concentration-dependent effects of Mg^{2+} on Gh-TDH nuclease activity.

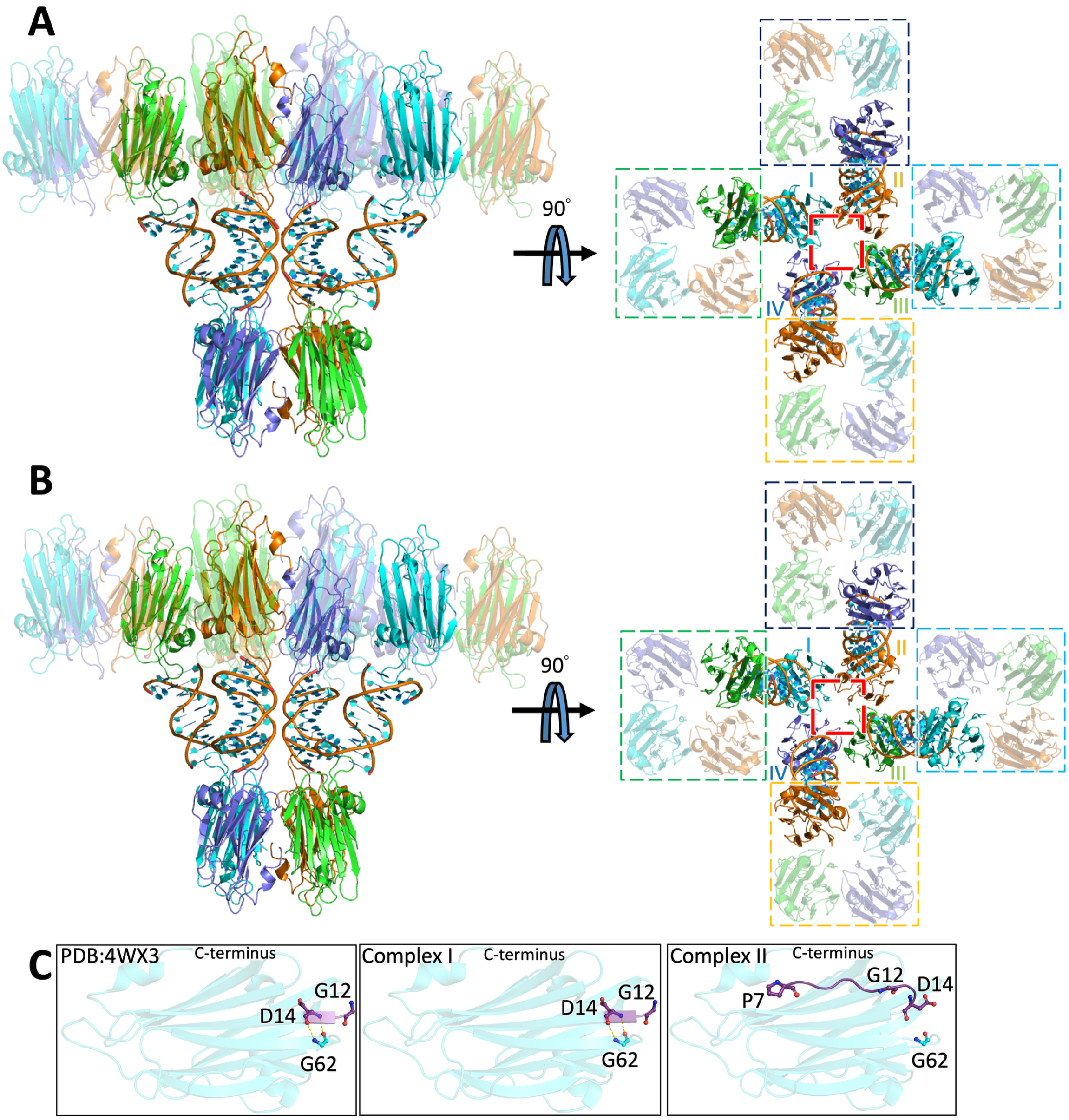

Fig. S7. Overall structures and symmetry mates of complexes I and II. Crystal structures of Gh-TDH-DNA complexes I and II. (A), Side (left) and top (right) views of complex I (right). (B), Side (left) and top (right) views of complex II (right). The Gh-TDH tetramer is indicated by large dashed boxes in dark blue, cyan, yellow, and green, and the N-terminal region (NTR) is highlighted by a small red dashed box. (C), Comparison of N-terminal conformations in Gh-TDH, complex I, and complex II, with residues Pro7–Gly12 shown in dark purple and G62 in cyan. Figures (A) and (B) show only one assembly in which a Gh-TDH tetramer bound to DNA. The upper Gh-TDH tetramers are depicted in transparent representation. The crystal packing of Gh-TDH and the symmetry-expanded view are provided in Supplementary Fig. 8.

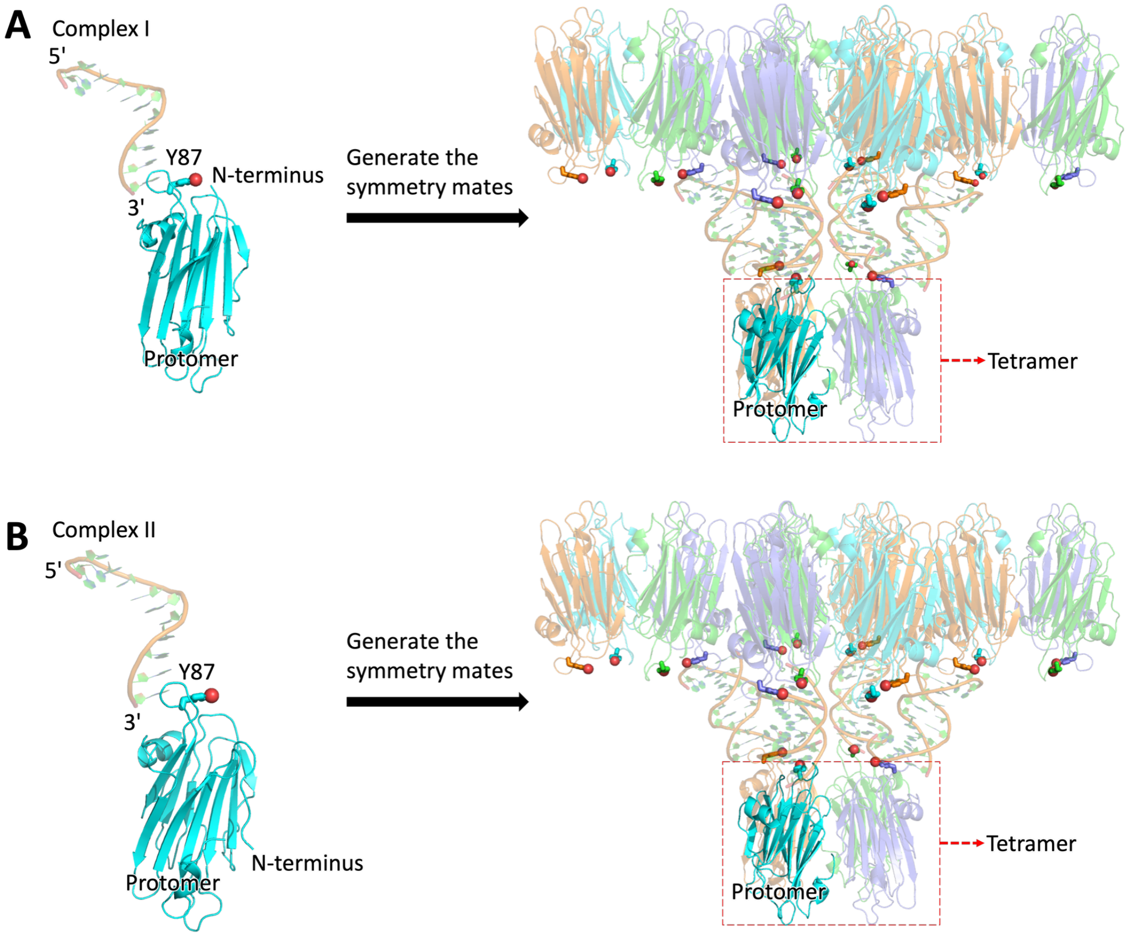

Fig. S8. Symmetry mates of complexes I and II. (A) Complex I and (B) Complex II. The left panels depict the molecules contained within a single unit cell, while the right panels display the assemblies generated by crystallographic symmetry. The non-transparent cyan protomer represents the original unit cell, while the surrounding transparent protomers illustrate the symmetry-expanded view forming the tetrameric assembly. Y87 is shown as sticks.

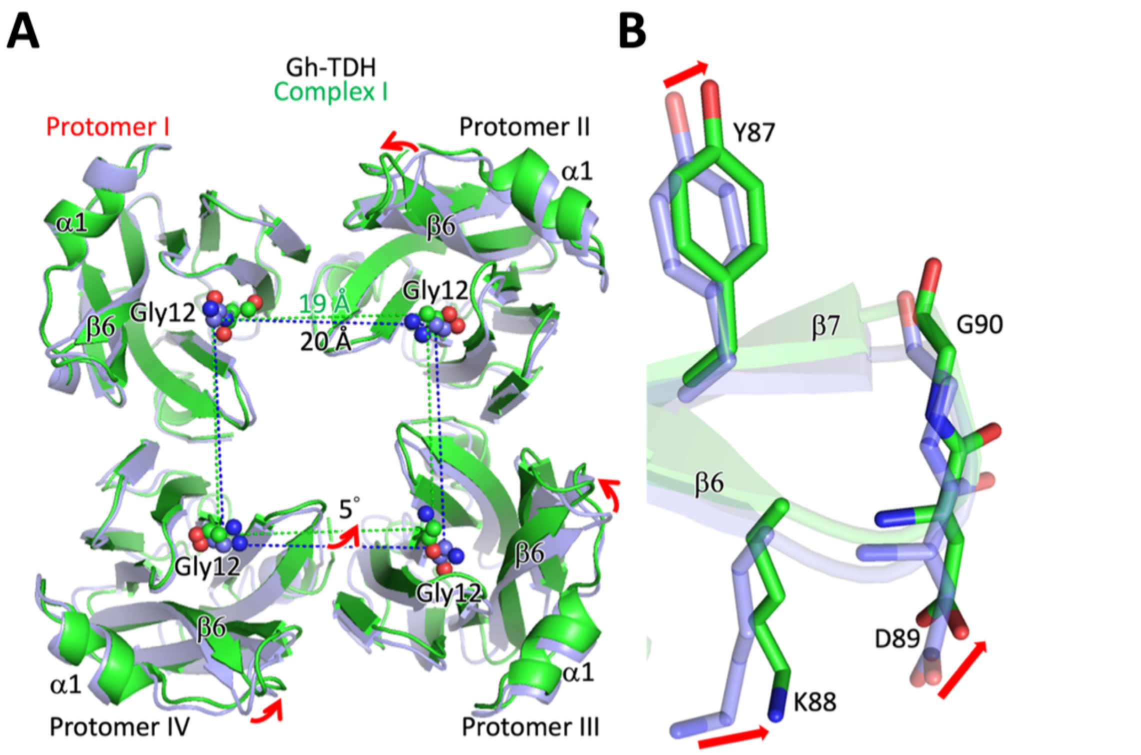

Fig. S9. Superposition of the Gh-TDH (blue, PDB entry: 4WX3) and complex I (green) structures. (A) The central pore size of both structures was calculated based on the distance of the α-carbon on Gly12 in the tetrameric Gh-TDH. The rotation axes of Protomer I in both Gh-TDH and complex I are highlighted in red text. The red arrow shows the counterclockwise rotation and conformational change of tetrameric Gh-TDH, taking the conformational change of β6 as an example. (B) Detailed view of the conformational change in the β-hairpin (^{87}YKDG^{90}) between Gh-TDH and complex I.

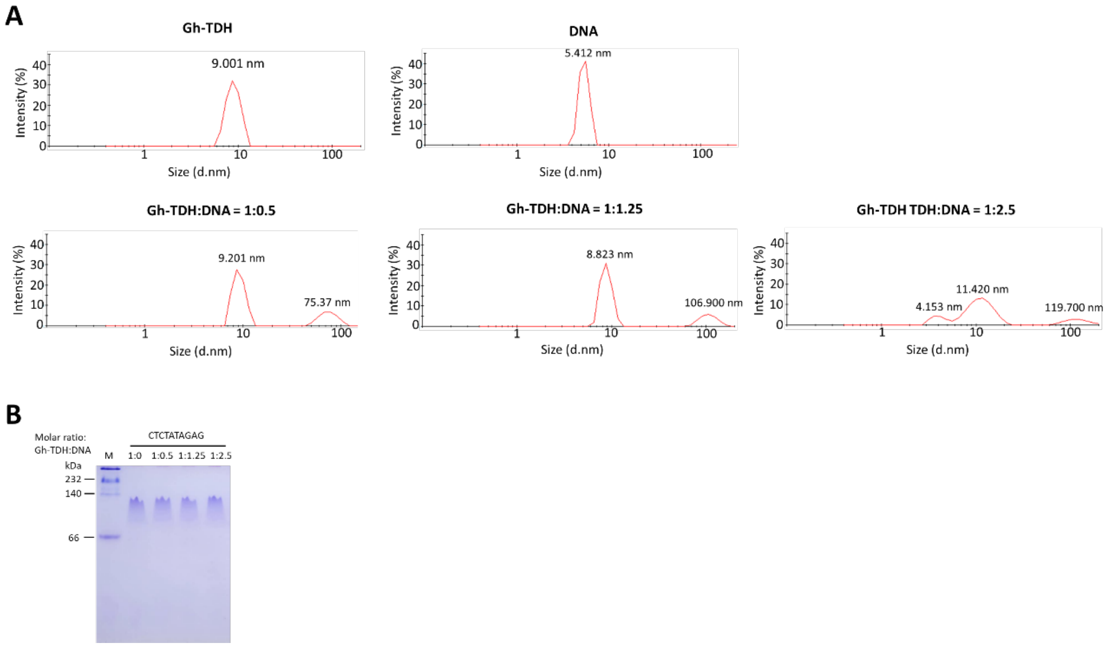

Fig. S10. Characterization of the solution state of Gh-TDH by DLS and native PAGE. (A) Dynamic light scattering (DLS) analysis of Gh-TDH, DNA substarte, and Gh-TDH–DNA mixtures at molar ratios of 1:0.5, 1:1.25, and 1:2.5. Upon addition of DNA substrate, a minor peak at ~100 nm is observed, likely arising from DNA-associated species. Notably, Gh-TDH remains predominantly as a ~10 nm species under all conditions tested, consistent with its tetrameric state. At a 2.5-fold excess of DNA substrate, an additional peak corresponding to free DNA becomes evident, while the Gh-TDH peak decreases in intensity and broadens, suggesting increased heterogeneity without disruption of the tetrameric assembly. (B) Native PAGE analysis of Gh-TDH in the presence of DNA at molar ratios of 1:0, 1:0.5, 1:1.25, and 1:2.5. Gh-TDH predominantly adopts a tetrameric state both in the absence and presence of DNA. High molecular weight native markers (Cytiva HMW Native Marker Kit) were used as size references.

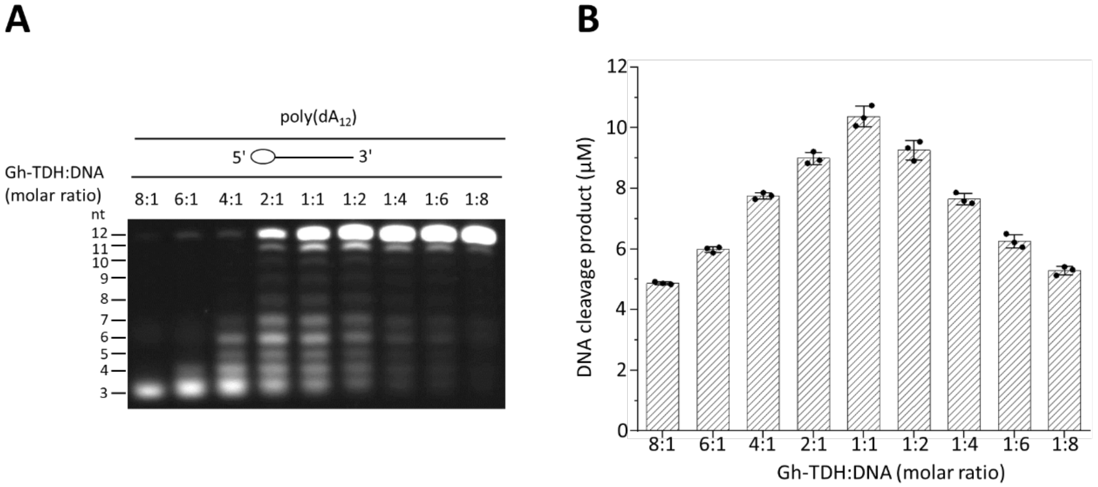

Fig. S11. DNA-binding stoichiometry of Gh-TDH. (A) Nuclease activity assays performed using different molar ratios of Gh-TDH and DNA substrate (poly(dA_{12})). The total concentration of Gh-TDH and DNA substrate was maintained at 5 μM, while the molar ratio of Gh-TDH to DNA was varied (8:1, 6:1, 4:1, 2:1, 1:1, 1:2, 1:4, 1:6, and 1:8). (B) Job plot analysis for determining the DNA-binding stoichiometry of Gh-TDH.

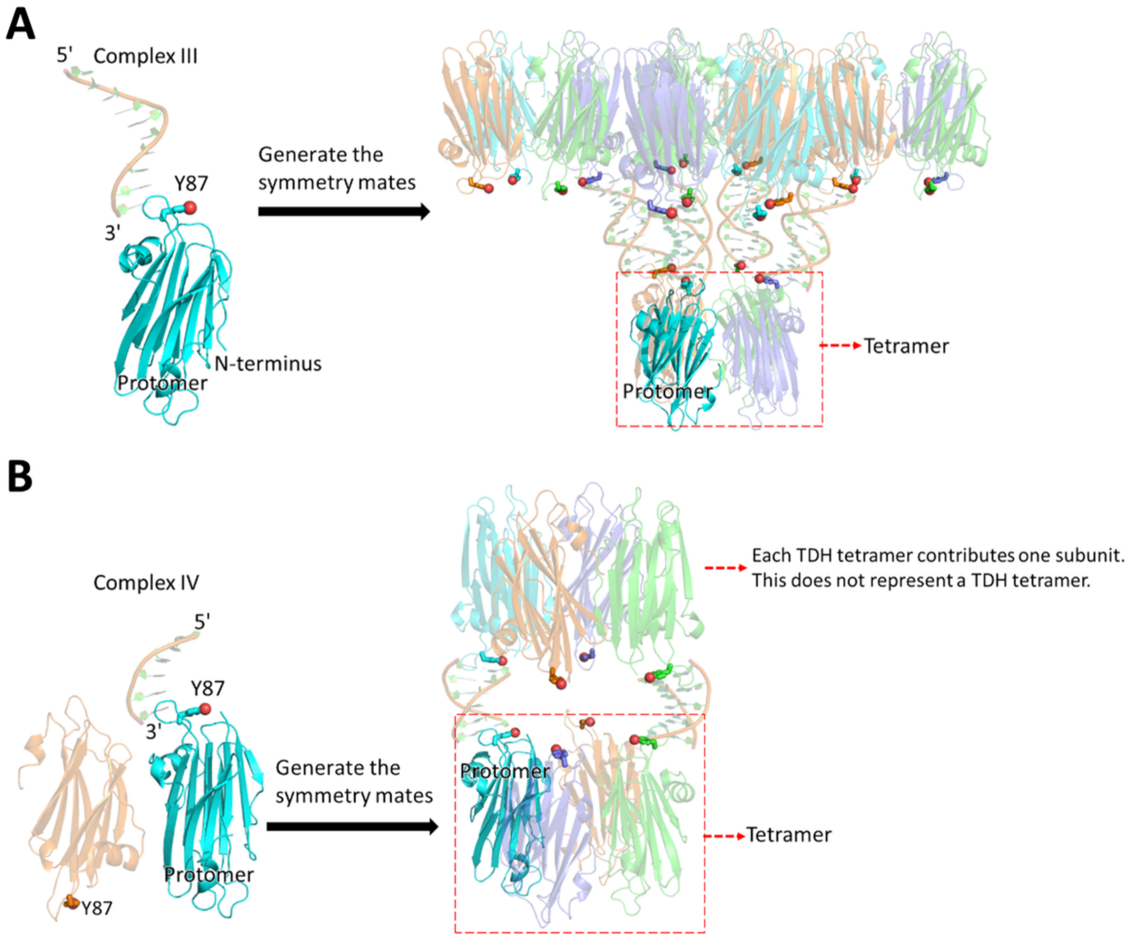

Fig. S12. Symmetry mates of complexes III and IV. (A) Complex III and (B) Complex IV. The left panels depict the molecules contained within a single unit cell, while the right panels display the assemblies generated by crystallographic symmetry. The non-transparent cyan protomer represents the original monomer within the unit cell, while the surrounding transparent protomers illustrate the symmetry-expanded view forming the tetrameric assembly. In the symmetry-expanded view of complex IV, the top side does not correspond to a Gh-TDH tetramer. Y87 is shown as sticks.

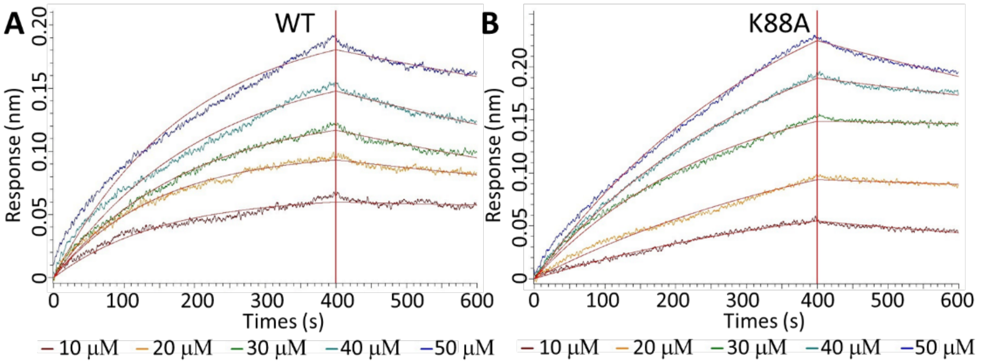

C

	K_D (μM)	K_D error	K_{on} (1/Ms)	K_{on} error	K_{off} (1/s)	K_{off} error	R^2
WT	14.8	1.60×10^{-7}	7.98×10^{1}	5.10×10^{-1}	1.18×10^{-3}	1.03×10^{-5}	0.9559
K88A	22.4	4.77×10^{-7}	3.49×10^{1}	5.10×10^{-1}	7.81×10^{-4}	1.21×10^{-5}	0.9909

Fig. S13. Kinetic binding curves of Gh-TDH variants with stem-loop dsDNA. (A), and (B), Binding curves of 5'-biotin stem-loop dsDNA with 4-nt 3'-overhang to Gh-TDH WT and the K88A variant. The x-axis represents the reaction time, while the y-axis indicates the BLI signal. The binding curves of five protein concentrations (10, 20, 30, 40, and 50 μM) are colored in brown, yellow, green, blue-green and dark blue, respectively. (C), The K_D, K_{on}, K_{off} value of stem-loop dsDNA with WT and the K88A variant.

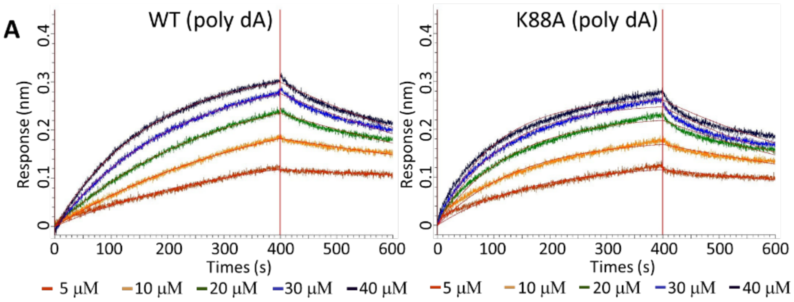

	K_D (μM)	K_D error	K_{on} (1/Ms)	K_{on} error	K_{off} (1/s)	K_{off} error	R^2
WT	2.61	4.17×10^{-8}	2.46×10^{2}	9.18×10^{-1}	6.42×10^{-4}	9.99×10^{-6}	0.9923
K88A	3.92	2.79×10^{-8}	3.76×10^{2}	1.2×10^{0}	1.47×10^{-3}	9.38×10^{-6}	0.9716

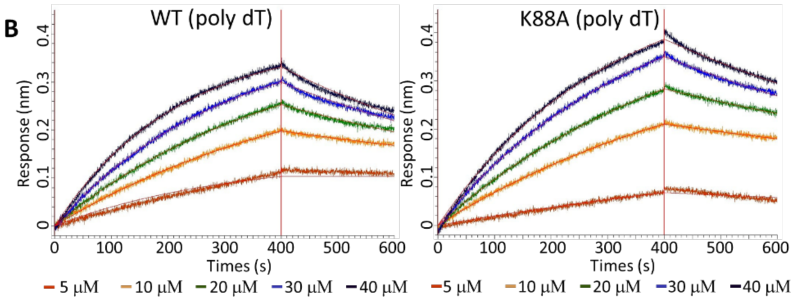

	K_D (μM)	K_D error	K_{on} (1/Ms)	K_{on} error	K_{off} (1/s)	K_{off} error	R^2
WT	2.39	5.02×10^{-8}	1.95×10^{2}	7.55×10^{-1}	4.67×10^{-4}	9.63×10^{-6}	0.9876
K88A	3.64	1.94×10^{-7}	1.41×10^{2}	1.76×10^{0}	5.12×10^{-4}	2.65×10^{-5}	0.9899

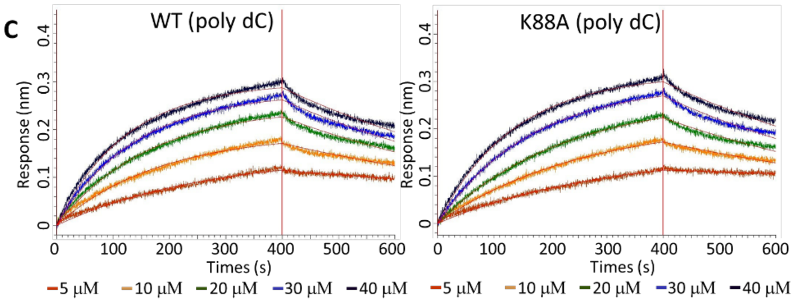

	K_D (μM)	K_D error	K_{on} (1/Ms)	K_{on} error	K_{off} (1/s)	K_{off} error	R^2
WT	3.96	3.78×10^{-8}	3.09×10^{2}	1.17×10^{0}	1.22×10^{-3}	1.07×10^{-5}	0.9853
K88A	5.01	4.30×10^{-8}	2.42×10^{2}	8.53×10^{-1}	1.21×10^{-3}	9.48×10^{-6}	0.9898

Fig. S14. Kinetic binding curves of Gh-TDH variants with ssDNA. Binding curves of 5'-biotin (A) poly(dA), (B) poly(dT), and (C) poly(dC) to Gh-TDH WT and the K88A variant, together with the K_D, K_{on}, K_{off} values. The x-axis represents the reaction time, while the y-axis indicates the BLI signal. The binding curves of five protein concentrations (5, 10, 20, 30, and 40 μM) are colored in red, orange, green, blue and black, respectively.

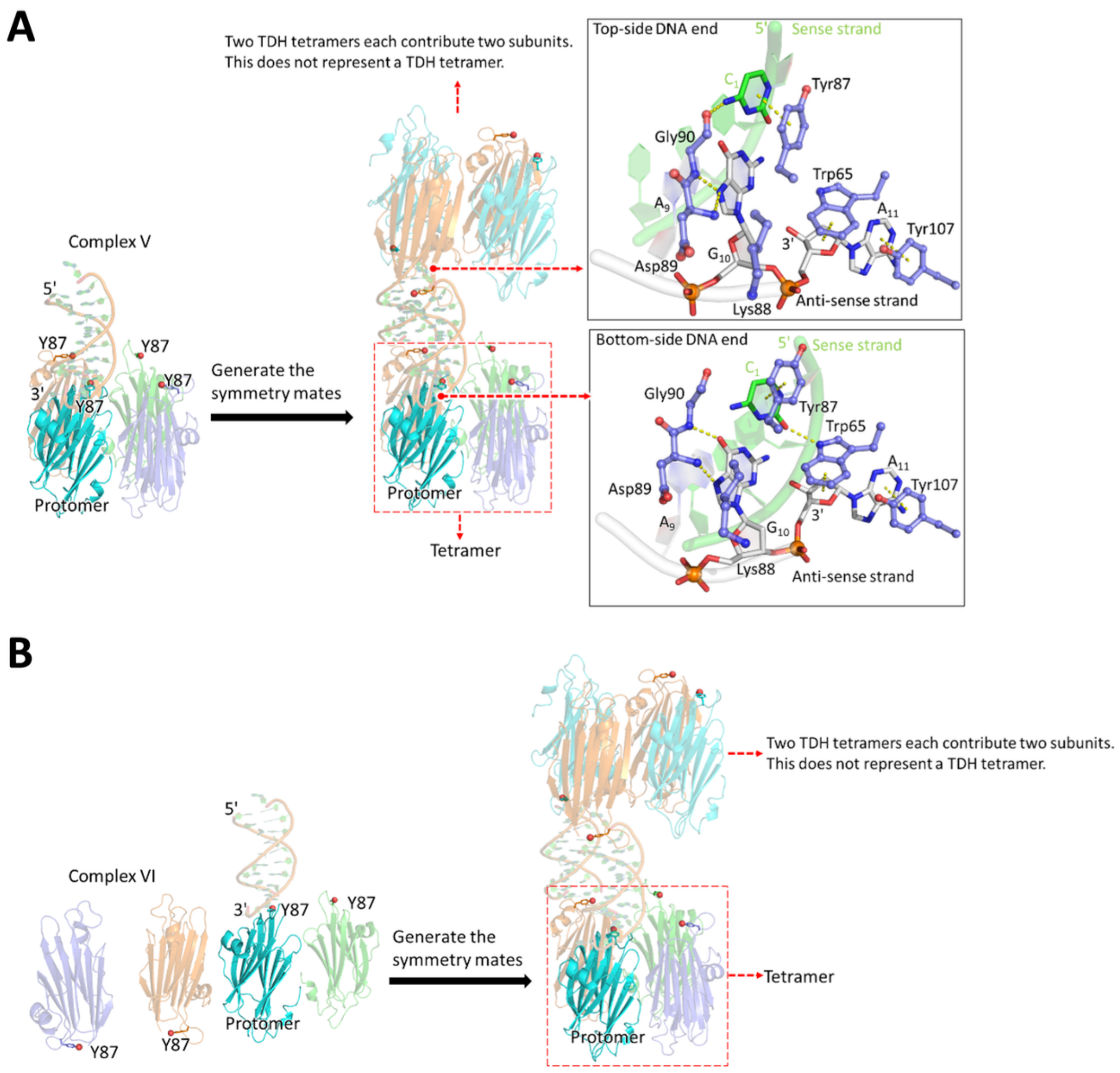

Fig. S15. Symmetry mates of complexes V and VI and binding mode of Complex V. (A) Complex V and (B) Complex VI. The left panels depict the molecules contained within a single unit cell, and the right panels display the assemblies generated by crystallographic symmetry. The non-transparent cyan protomer represents the original protomer within the unit cell, while the surrounding transparent protomers illustrate the symmetry-expanded view forming the tetrameric assembly. In the symmetry-expanded views of complexes V and VI, the top side does not correspond to a Gh-TDH tetramer. In complex V, the local view reveals two distinct DNA-binding modes at the top-side and bottom-side DNA ends.

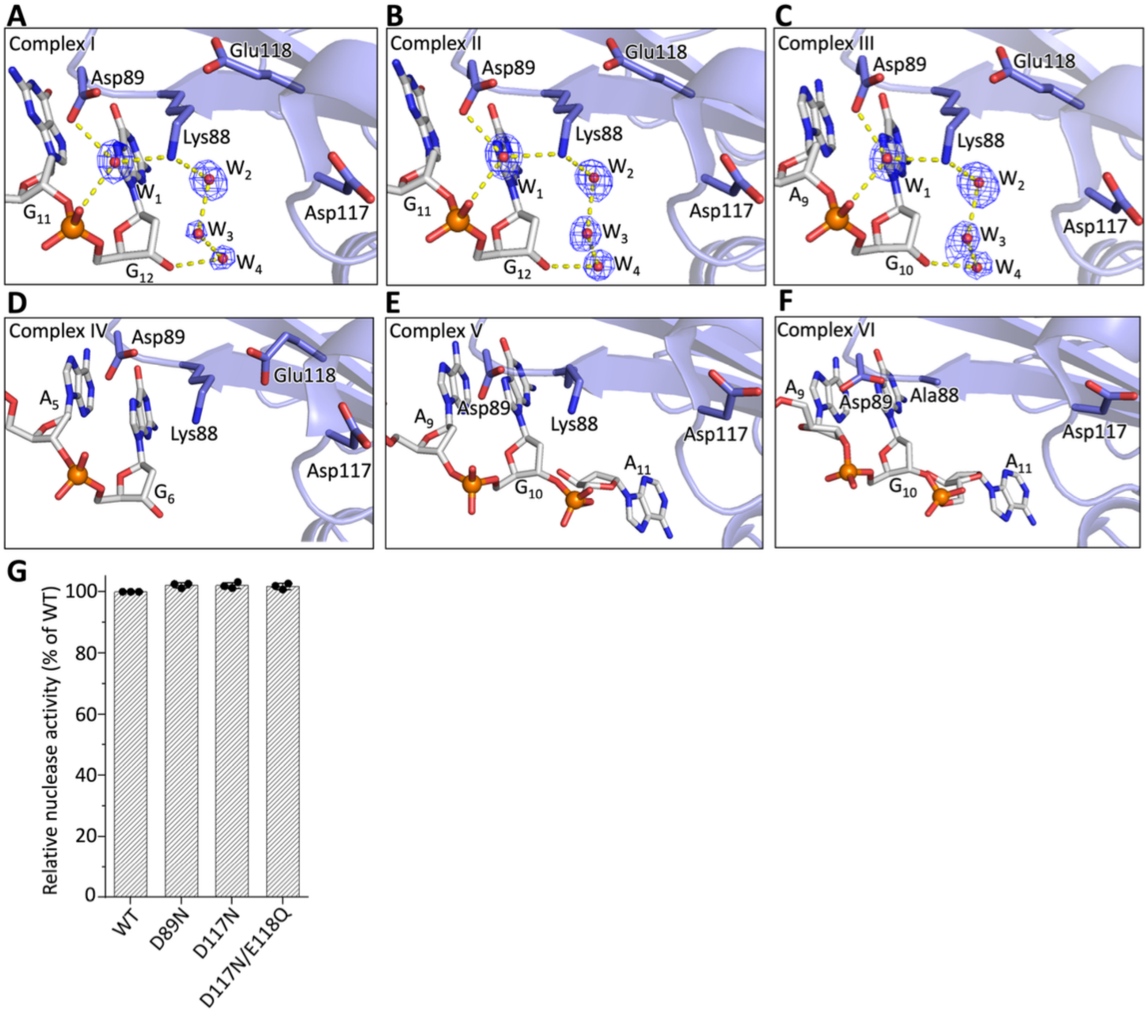

Fig. S16. Active-site water molecules and nuclease activities of selected variants. Local view of the proposed active site in (A)–(F), complexes I–VI. DNA are displayed in white sticks. Putative residues involved in DNA cleavage are shown in blue sticks. The cartoon is in a transparent mode. The omitted electron density maps ($2F_o{-}F_c$, 1.0 σ) of the water network for W_1, W_2, W_3, and W_4 are shown as blue mesh. (G) Mutagenesis analysis of the Gh-TDH variants. Relative activities of Gh-TDH variants (D89N, D117N, and D117N/E118Q) were compared with Gh-TDH wild-type in digesting 5'-FAM stem-loop dsDNA with 4-nt 3'-overhang. Each of the activity assays was performed in triplicate (n=3) and the data were presented as mean value ± standard error of the mean (SEM).

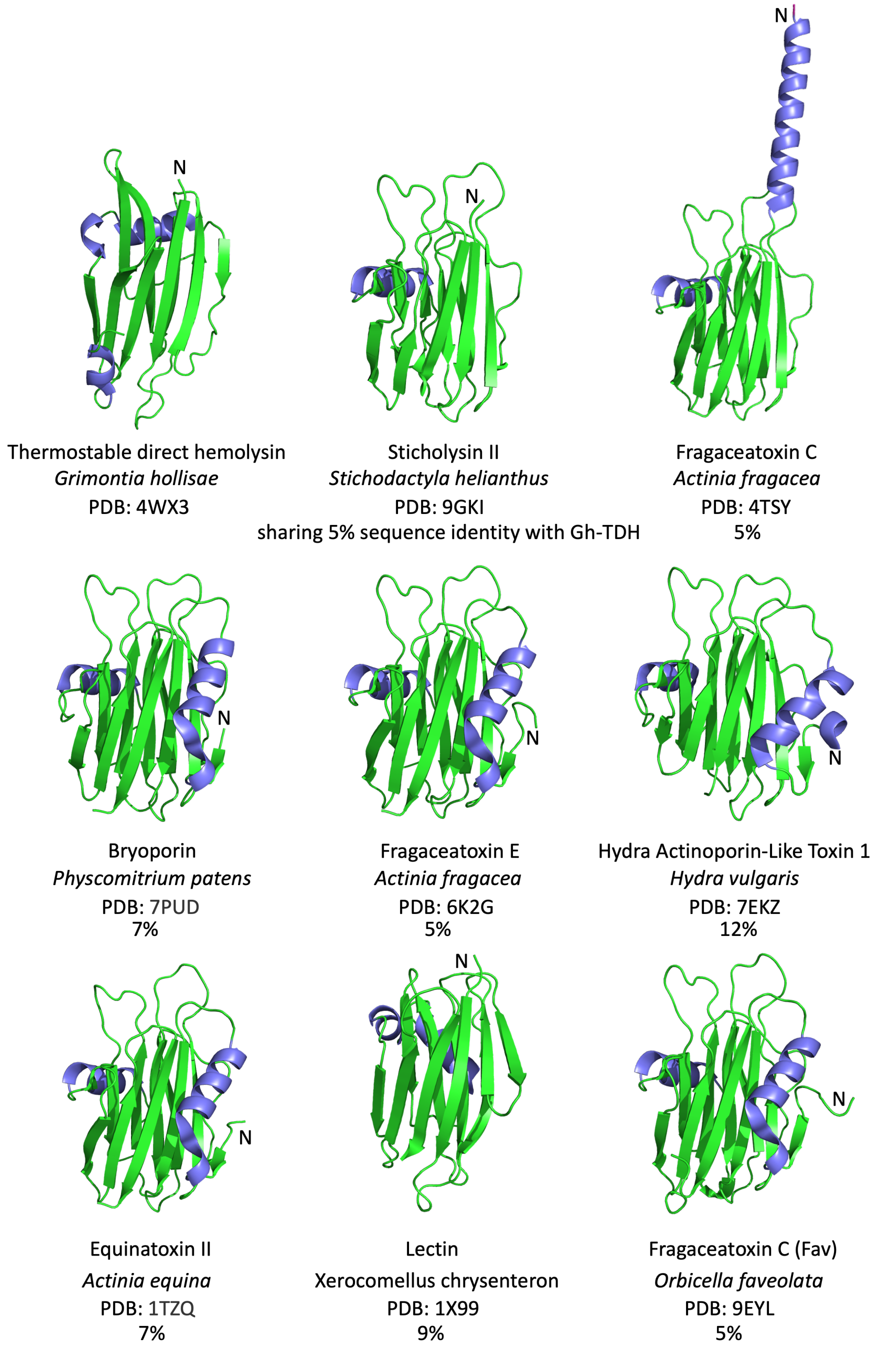

Fig. S17. Structural comparison of Gh-TDH with homologous proteins identified by DALI analysis. Gh-TDH is compared with eight structural homologues: Sticholysin II (Z = 11.6), Fragaceatoxin C (Z = 11.1), Bryoporin (Z = 11.0), Fragaceatoxin E (Z = 10.4), Hydra actinoporin-like toxin 1 (Z = 10.1), Equinatoxin II (Z = 10.1), lectin (Z = 10.0), and Fragaceatoxin C (Fav) (Z = 9.9). Among these homologues, all proteins except lectin belong to the pore-forming toxin (PFT) family. Secondary structure elements are colored with β-sheets in green and α-helices in blue. The numbers shown below each structure indicate the percentage of sequence identity relative to Gh-TDH.

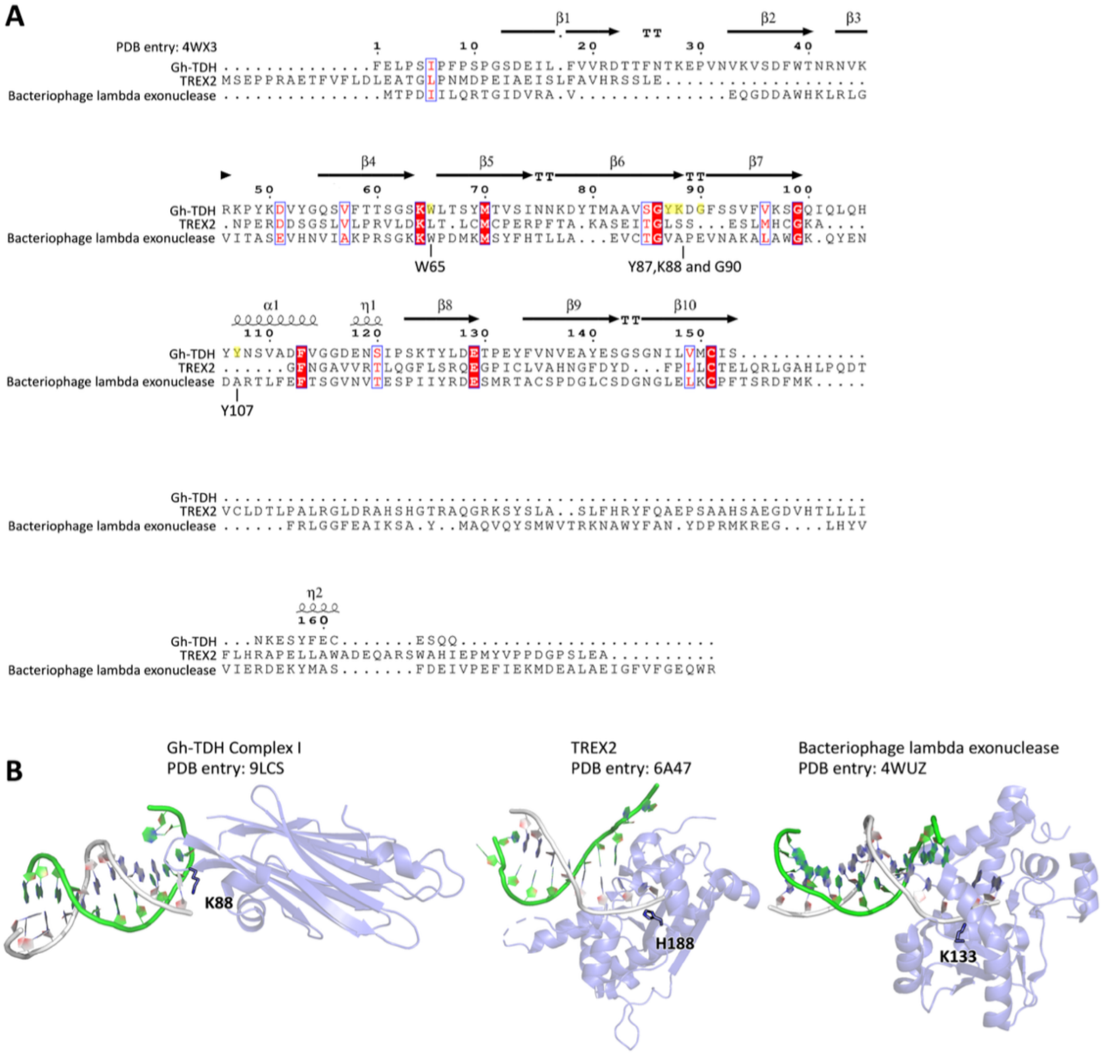

Fig. S18. Sequence alignment and structural comparison of Gh-TDH with representative nucleases from the DEDD and DEK superfamilies. (A) Sequence alignment of Gh-TDH with the DEDD superfamily enzyme TREX2 from mouse (NP_036037.1) and the DEK superfamily enzyme bacteriophage lambda exonuclease from *Lambdavirus lambda* (WP_000186853.1). Residues in Gh-TDH associated with nuclease activity and DNA binding are highlighted in yellow. Aligned residues are colored on the basis of the level of conservation (red background with white characters or red characters with bold font shows strict identity, red characters for similarity, and blue frame for similarity across group). The secondary structure of Gh-TDH (PDB entry: 4WX3) is depicted above the sequence alignment. (B) The crystal structures shown correspond to Gh-TDH, TREX2, and bacteriophage lambda exonuclease, each in complex with DNA. The residues K88 in Gh-TDH, H188 in TREX2, and K133 in bacteriophage lambda exonuclease, are key catalytic residues involved in DNA cleavage. "TT" denotes a turn in the protein secondary structure.

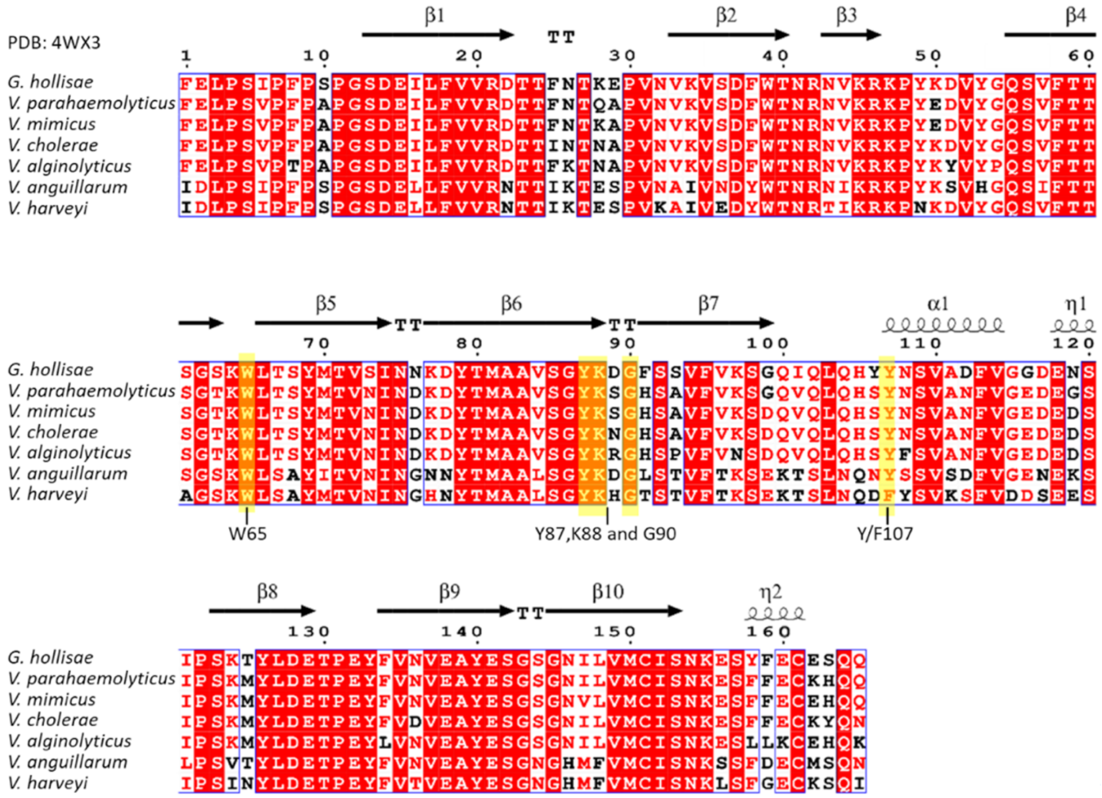

Fig. S19. Sequence alignment of Gh-TDH with selected homologues. The Gh-TDH (WP_040528653.1) homologues are selected from *V. parahaemolyticus* (WP_005463268.1), *V. mimicus* (WP_158139425.1), *V. cholerae* (AAA27545.1), *V. alginolyticus* (ABD96016.1), *V. anguillarum* (ACS74755.1), and *V. harveyi* (WP_080540244.1). Conserved residues involved in nuclease activity and DNA binding are highlighted in yellow. Aligned residues are colored based on the level of conservation (red background with white characters or red characters with bold font shows strict identity, red characters for similarity, and blue frame for similarity across group). “TT” denotes a turn in the protein secondary structure. The secondary structure of Gh-TDH (PDB entry: 4WX3) is shown above the sequence alignment.

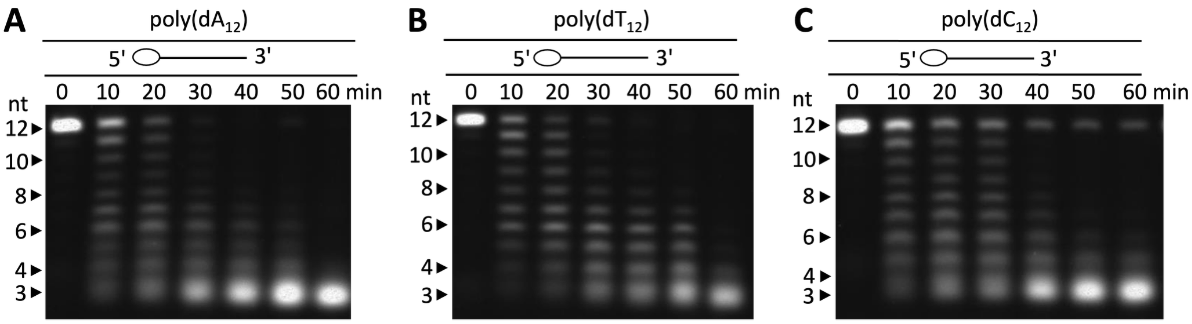

Fig. S20. Nuclease activity assay of Vp-TDH. (A) Nuclease activity of Vp-TDH digested poly(dA_{12}). (B) Nuclease activity of Vp-TDH digested poly(dT_{12}). (C) Nuclease activity of Vp-TDH digested poly(dC_{12}). 10 μM Vp-TDH and poly(dN_{12}) were incubated at 37 °C for 60 min, with gel samples taken every 10 min. All ssDNA substrates were cleaved by Vp-TDH down to fragments of 3 nucleotides.
